# Imaging referral guidelines in Europe: now and in the future—EC Referral Guidelines Workshop Proceedings

**DOI:** 10.1007/s13244-013-0299-8

**Published:** 2013-12-13

**Authors:** Denis Remedios, Monika Hierath, Nick Ashford, Mario Bezzi, Peter Cavanagh, Jean-François Chateil, Philippe Grenier, Georgi Simeonov, Valerie Vilgrain

**Affiliations:** 1 Northwick Park Hospital, London, UK; 2 European Society of Paediatric Radiology (ESPR), Vienna, Austria; 3 Royal College of Radiologists (RCR), London, UK; 4 Cardiovascular and Interventional Radiological Society of Europe (CIRSE), Vienna, Austria; 5 French Society of Radiology (SFR), Paris, France; 6 European Commission Directorate-General for Energy (EC DG Energy), Brussels, Belgium

## Abstract

As an integral part of the European Commission (EC) Imaging Referral Guidelines Project a 1.5-day workshop was held in Vienna on 20–21 September, 2012. At this workshop, models and good practices regarding the appropriateness and use of imaging referral guidelines (Guidelines) in Europe and worldwide were presented, together with the results of a survey of Guidelines in Europe. The latter included ideas, innovations and wishes for future Community action.

Recommendations for future Community action:

- Stronger measures should be taken by the EC and the European competent authorities for making Guidelines available and used in all EU member states.
- Evidence-based Guidelines with separate guidance for children should be issued or endorsed by a trusted European organisation.
- Educational initiatives and electronic requesting in connection with clinical decision support (CDS) systems should be used to improve the implementation of Guidelines.
- Monitoring of Guidelines implementation and use should be by clinical audit, particularly external audit, but also by local/internal audit.

**Electronic supplementary material:**

The online version of this article (doi:10.1007/s13244-013-0299-8) contains supplementary material, which is available to authorised users.

## Introduction

Over 60 participants attended the workshop in Vienna [1], with registration of representatives from national radiology societies and representatives from the regulatory bodies of 30 European countries. Speakers from Europe, USA, Canada and Australia included expert advisors from the World Health Organisation (WHO), International Atomic Energy Agency (IAEA) and European Association of Nuclear Medicine (EANM), as well as key stakeholders such as representatives from patient groups, radiographer societies and general practitioners.

The programme included over 30 talks, divided into five sessions. Each session allowed ample time for discussions, with enthusiastic participation from the floor.

## Session summaries

The Workshop’s sessions each dealt with a specific subject within the area of Imaging Referral Guidelines in Europe.

Session 1, *Scene Setting*, served as an introduction to the topic and gave an overview on the current status of Referral Guidelines in Europe. Experts from International Organisations such as the WHO and IAEA presented their perspective and invited speakers from Europe, USA, Canada and Australia shared their views on Referral Guidelines from a national perspective. This session covered the drivers for the effective use of Guidelines, followed by the experience from a number of countries who had developed and used their own referral guidelines. The key messages included the following:

### Drivers for the effective use of Guidelines

European and International Legislation related to radiation protection requires effective justification of imaging tests involving radiation based on evidence-based guidance [2]. In addition there are concerns regarding increasing per capita dose from medical imaging across Europe and internationally. On the positive side, earlier access to appropriate imaging is known to affect outcomes in a number of conditions.

### Production of Guidelines

There is a similar process for the production and formatting of Guidelines by those countries who presented their experience [3–5], with the exception of Western Australia [6]. Most countries displayed the Guidelines in a tabular form involving a grading system but in Western Australia the guidance was integrated into a decision-making flow-chart algorithm. All countries reported that the production of evidence-based Guidelines was resource intensive, particularly in regard to radiologist input into the process.

There was acknowledgement of duplication of effort, with the same evidence being reviewed in each individual country reaching the same or similar conclusions. The ownership and development of Guidelines by radiologists may well have an impact on the adoption of, and compliance with, Guidelines by the end-user clinician.

### Challenges in evidence

The evidence base is often related to comparison of differing modalities in terms of accuracy, safety and efficiency, but there are less robust data on the impact of the use of imaging to patient outcomes. Improvements in process are often considered as a proxy for outcome. Evidence in many pathways is not always present and, if present, is not always high level, widely applicable or robust. A key factor should be the overall efficiency gains within the health system by the correct use of Guidelines.

### Why don’t Guidelines work?

There were common challenges and barriers at the following stages: awareness, agreement, adoption and adherence. Specific barriers that were discussed included:

- The total number of Guidelines was seen as a potential barrier for adoption/adherence—often in excess of 300.
- It was agreed that there had to be ease of access by the end user at the time of referral, and for this reason paper versions of the Guidelines were the least effective. Web-based or App-based Guidelines should in principle improve this.
- Ideally Guidelines should be free to recognised referrers but this requires a stable financial model for their production.
- Guidelines were often regarded as being owned by the radiology department or radiologists, with no real ownership by the referrer. In some instances certain clinical specialties had developed their own guidance as part of clinical pathways which had the potential to conflict with the radiology guidelines.
- The increasing concern by referrers of the potential for litigation if they miss a diagnosis leads to unnecessary overuse of imaging.
- Patient expectations are continuing to increase and their insistence on imaging can over-ride clinical judgement.

### Solutions

Potential solutions to the barriers and challenges were suggested as follows:

- European or International collaboration on reviewing evidence.
- Focusing on fewer Guidelines which are likely to have the biggest impact on safe, quality and efficient care.
- Involve referrer groups in the production and promotion of Guidelines.
- Integrating Guidelines into the electronic referral process—‘decision support’ software.
- Increasing dose awareness as part of the guideline process.
- Improved communication/promotion.
- Focus on improving the evidence base for the Guidelines that are likely to have the most impact.
- Production of internationally agreed Guidelines with the ability to customise to national/local circumstances.
- Education/training/audit.

Session 2, *Specific Issues*, addressed the perspectives of stakeholders from paediatrics, interventional radiology and nuclear medicine, as well as representatives from radiographer societies and patient groups.

This session gave an excellent overview of referral guidelines from the perspectives of varying and diverse stakeholders in respect to Guidelines with input from:

- Paediatric radiology
- Interventional radiology
- Nuclear medicine
- Radiological protection, both dose issues and risk as well as implementation issues
- Radiography and the radiographer
- General practitioners
- The patient’s perspective

Not surprisingly all speakers were strongly in favour of Guidelines although each speaker saw and described their structure, design and use with differing perspectives.

The key presentation relating to paediatrics stressed the greater sensitivity of children to radiation-induced effects and the longer period for effects to be manifested. These risks were far greater with CT and fluoroscopy, rather than radiography. Education is essential and the development and provision of specific guidelines for children is essential. Discussion centred on whether these should be stand-alone Guidelines or separate guidelines integrated into a unified publication.

Interventional radiology is a rapidly changing field hence the need for frequent updates with practice guidelines for interventional radiology not fully implemented in current diagnostic radiology guidelines.

Interventional radiologists act as clinical gatekeepers ensuring that the design of Guidelines involves multidisciplinary practice, collaboration of a large number of diverse stakeholders and robust education, not only across the healthcare community but also to patients, the public and media.

It was noted that procedural risks in interventional radiology were often far higher than radiation-related risks.

Nuclear medicine benefits from fewer procedures and Guidelines are committee driven and jointly written with clinical specialities. Interestingly technologists prepare parallel Guidelines. All requests are routinely checked and there is a strong culture of audit.

The balance between risk and benefit is pivotal, the assessment of dose alone is not helpful and justification requires knowledge of clinical circumstances. The wording of Guidelines must be carefully chosen, especially related to possible risk of misuse, criteria and appropriateness.

Key points of implementation are that Guidelines are legally required, but more importantly that they are useful, should be kept up to date, and should be used and audited and their development funded.

Radiographers were portrayed as playing a key role at the interface between patient and technology, being the pivot between referrers, patients and radiologists. They are therefore a key player in the implementation of referral guidelines.

A presentation from a general practitioner stressed that excellent communication between the patient, general practitioner and radiologist is essential. This will be facilitated by clear referral guidelines.

The patient’s perspective stressed the need for dose information, clear communication (no acronyms), the need to be sensitive to the patient’s needs, and that the patient is a member of the team—“No decision about me without me”.

The varying and diverse interpretation of “Specific Issues” demonstrates the need for a broad base of knowledge expertise and experience when designing and implementing Guidelines.

Session 3, *Survey Feedback*, dealt with the formulation and findings from the survey of Guidelines in Europe, conducted as an earlier part of the EC Referral Guidelines Project. Within this session workshop participants were given an opportunity to present examples of good practices from their own countries followed by enthusiastic discussion.

The survey feedback summarised the survey. It was web-based and involved multiple organisations from 30 countries. These included radiology and nuclear medicine societies and competent authorities. The questions were comprehensive and related to responsibilities, format, circulation and purpose of the referral guidelines. In addition, the survey participants were asked to share their views on barriers to implementation and their potential solutions. In establishing the survey, consideration was given to ease of understanding, completion and subsequent analysis.

The analysis provided a wealth of data. More significant information related to awareness of the legal requirement for Guidelines (60 %) and the difference between this requirement and actual availability. There was variation as to the origin of the Guidelines in each of the countries—some were based on practice within the Member State, while others were heavily or entirely based on the publications from organisations external to their country (e.g. translation of RCR guidelines). The survey provided information on a number of key elements and factors of the Guidelines including the evidence base, the grading of procedures and the associated radiation dose.

Session 4 dealt with *Innovations* for improvement in Guideline use. Examples of innovations in use worldwide and potential solutions drawn from other areas of medicine were discussed.

The need was identified to improve Guideline development with specific emphasis on the level of Evidence (currently approximately 30 % of Guidelines are not evidence-based). Guidelines should be informed by secondary research and particularly by systematic reviews where available. There was agreement for the need of European Guidelines, either as a synthesis of several National Guidelines or developed by European bodies.

Suggestions made for Guideline development include:

- An imaging specialist should be the author.
- Guidelines should be aimed at referrers.
- Guidelines should also be of use to patients and the public.
- Guidelines should be included in the curriculum of medical undergraduates and residents.

The plan for the future should include developments to electronic requesting (computerised patient order entry, CPOE) in connection with CDS systems.

The suggestion was made for developing selected high-impact Guidelines using simple, fast and transparent software, under the leadership of medical practitioners and validated in small integrated patient groups by motivated physicians.

Web-based guidance and adequate resources are required. In addition, tablet and smart phone applications (apps) would be useful.

## Workshop conclusions and recommendations

Following presentations from over 30 speakers and lengthy discussions at the European Workshop, Work Package 2 of the EC Guidelines Project, a clearer picture of the current situation has emerged with a coherent message as to the way forward.

### Guideline availability and use in Europe

Data to inform the availability of referral guidelines are not as straightforward as hoped. The survey has shown discordance of responses between some national competent authority and professional society representatives, possibly related to issues of awareness of Guideline existence and the legal requirement for Guidelines. In addition, there are issues of access to national guidelines, which may be related to format or media.

In the European survey [7], respondents in two-thirds of European countries know of the legal requirement for Guidelines nationally, and in these countries two-thirds of these have Guidelines available. Of the one-third of European countries where there is no awareness of a legal requirement, only one-third have Guidelines available. Discussion at the Workshop identified that several countries had work in progress to update or adopt Guidelines, with considerable progress since the survey took place, 4 months previously.

A further, brief, follow-up survey to ascertain the up-to-date status of Guideline availability was planned following the Workshop to enable the current work in progress to be reflected in the final report.

### Guideline implementation

Workshop participants agreed that the availability of Guidelines did not equate with their use. As Guidelines cannot encompass all clinical scenarios, adherence can only be expected in up to 90 % of clinical scenarios. Participants at the Workshop reported that even where Guidelines are available, their uptake and use are limited. Reasons for this may include: sub-optimal clinician buy-in, conflicting advice from other (often clinical) guidelines, and the difficulty associated with monitoring and encouraging Guideline use.

Tools which can encourage Guideline use include:

- *Education*—at all levels of training and continuing professional development. Web-based, training courses and inclusion in curricula as well as educational reminders [8, 9]. These initiatives are already in progress [10].
- *CDS systems*, particularly those with flexible architecture to fit existing requesting systems. Examples of CDS systems were available from many speakers and there was great interest amongst participants, both radiologists and regulators.
- *Management systems*, e.g. Radiological benefit management.
- *Governmental incentives*, e.g. Payment for performance [11], Quality Outcome Framework [12].

### Monitoring of Guidelines

Measures for reinforcement of Guidelines were considered essential to provide encouragement for use and to ensure sustained benefit. The European survey identified models, particularly external clinical audit as the preferred means, with a secondary role for local internal audit. Self-regulation [13, 14] by health professionals and inspection had less support.

### Guideline development

Areas of agreement for Guideline development methodology which were identified from the European survey and consolidated at the Workshop are:

- Preference for European Guidelines, which may best be adapted and amalgamated from mature, trusted, nationally developed evidence-based guidelines. This option was considered to be more practical and expeditious than the centralised development of European Guidelines de novo. Later iterations of European Guidelines should encompass a more inclusive methodology with consensus from experts from multiple European countries.
- Need to include dose information in a form understood by referrers and patients.
- Separate section for imaging of children.
- Stakeholders, including referrers and patient representatives, should be involved.
- Recommendations based on evidence of efficacy, radiation safety and cost.

## Electronic supplementary material

Below is the link to the electronic supplementary material.

Supplementary Material 1 Workshop proceedings, Session 1 *Scene Setting* (PDF 2840 kb)

Supplementary Material 2 Workshop proceedings, Session 2 *Specific Issues* (PDF 989 kb)

Supplementary Material 3 Workshop proceedings, Session 3 *Survey Feedback* (PDF 110 kb)

Supplementary Material 4 Workshop proceedings, Session 4 *Innovations* (PDF 714 kb)
