# Supplementary material for: Imaging referral guidelines in Europe: now and in the future—EC Referral Guidelines Workshop Proceedings
Source: Insights Imaging. 2013 Dec 13;5(1):9–13. doi: 10.1007/s13244-013-0299-8 (PMC3948903; doi:10.1007/s13244-013-0299-8)
Supplement: Supplementary file 1 — Workshop proceedings, Session 1 Scene Setting (PDF 2840 kb) [file 13244_2013_299_MOESM1_ESM.pdf]

Project ENER/D4/315-2011
Implementation of Council Directive 97/43/Euratom requirements concerning referral criteria for medical imaging in the European Union

IMAGING REFERRAL GUIDELINES IN EUROPE: NOW AND THE FUTURE. EC REFERRAL GUIDELINES WORKSHOP Billrothhaus, Vienna. September 20-21 2012

Workshop Proceedings

Session 1: Scene Setting

Chair: Myriam Hunink, European Society of Radiology (ESR)
Rapporteur: Pete Cavanagh, Royal College of Radiologists (RCR)

Talk 1: Scene Setting

Denis Remedios, European Society of Radiology (ESR) Project Lead

Dr. Denis Remedios
Northwick Park Hospital, Harrow, UK
denis.remedios@imperial.ac.uk

Introduction

The Project ENER/D4/315-2011, Implementation of Council Directive 97/43/Euratom requirements concerning referral criteria for medical imaging in the European Union fulfils the obligation of Member States "to ensure that recommendations concerning referral criteria for medical exposures, including radiation doses, are available to the prescriber of medical exposures". (Article 6.2 of Council Directive 97/43/EURATOM (Medical Exposures Directive, MED) [1]

Based on this requirement, a number of Member States have developed national referral guidelines for clinical imaging as guidance for the referring physicians to justify radiological imaging procedures and to ensure the highest possible safety of patients when submitted to radiation exposure. Imaging referral guidelines (Guidelines) have been available in Europe since 1989 when the Royal College of Radiologists (RCR) first published "Making the best use of a department of clinical radiology" [2]. The Radiation Protection 118 Referral Guidelines for Imaging (RP 118) [3] were published in 2000 by the European Commission, (based on the Royal College of Radiologists 1998 publication "Making the best use of a department of

clinical radiology: guidelines for doctors”). The French Society of Radiology (SFR) published imaging referral guidance in 2005 and updated in 2013, “Guide du bon usage des examens d'imagerie médicale” [4]. Rapid developments in imaging technology and new advances in medical imaging required an update of the guidelines by the European Commission in 2003.

The value of evidence-based Guidelines for justification at International Commission on Radiological Protection (ICRP) level 2 [5] and reduction of unhelpful medical exposures was shown in early studies [6, 7]. Such guidance is also helpful to promote good medical practice and may improve cost effectiveness by encouraging the best test first.

The proposal for the project “Implementation of Council Directive 97/43/Euratom [6] requirements concerning referral criteria for medical imaging in the European Union” was submitted as a consortium of several partners in June, 2011. Partner organisations are:

- European Society of Radiology (ESR) http://www.myesr.org/
- Royal College of Radiologists (RCR) www.rcr.ac.uk/
- French Society of Radiology (SFR) www.sfrnet.org
- Cardiovascular and Interventional Radiology Society of Europe (CIRSE) http://www.cirse.org/
- European Society of Paediatric Radiology (ESPR) http://www.espr.org/

Members of the steering committee are:

- Denis Remedios, ESR, chair
- Monika Hierath, ESR project manager
- Peter Cavanagh, RCR (and Nick Ashford)
- Philippe Grenier, SFR (and Valerie Vilgrain)
- Mario Bezzi, CIRSE
- Jean-François Chateil, ESPR (and Karen Rosendahl)
- Georgi Simeonov, EC representative

The overall aim of this project is to review the situation in European Union (EU) Member States regarding the fulfilment of their obligations under MED Article 6.2.

The full project comprises 3 main tasks:

1. the conduct of an EU-wide study on the availability, development and implementation of referral guidelines for radiological imaging in the EU Member States,
2. the organisation of a European Workshop with relevant representatives from the EU Member States, and
3. the development of conclusions of the workshop regarding the need for national and/or Community action.

Related international projects

World Health Organization (WHO)

- Global Initiative on radiation safety in health care settings launched in 2008 [8]. Sixty-seven participants, including experts from 25 countries and representatives from 15 international organisations, professional associations and scientific societies, have agreed to collaborate in this initiative. Based on the collected feedback the global

strategy was developed; main activities were identified under three areas of work: risk assessment, risk management and risk communication; ways for enhancing collaboration and engaging key stakeholders were proposed; and a roadmap was outlined.

- Guidelines consultancy 2010 [9]. Key representatives of the world's leading medical imaging societies have recommended that a common set of global referral guidelines for appropriate use of medical imaging be produced, in the first such global meeting of experts convened under WHO auspices in nearly two decades.

International Atomic Energy Agency (IAEA)

- International Workshop on Justification of Medical Exposure, Brussels 2009 [10]. This Workshop organised by the IAEA with the EC, addressed: referral guidelines; communication and risk; audit & justification; and special problems.
- Triple A, "Awareness, appropriateness and audit", 2010 [11]. Clinical audit was regarded as a key tool in ensuring that justification becomes an effective, transparent and accountable part of normal radiological practice. Justification would be facilitated by the "3 As": awareness, appropriateness and audit.

Awareness: The need for guidelines

There is wide recognition that imaging referral guidelines are needed to support decisions for imaging investigations globally. This is driven by the following key points:

- Diagnostic radiology in USA now accounts for almost as much radiation as natural causes (15% in 1980 to 48% in 2006) [12] (Fig. 1).
- CT exams have increased at 10% pa in USA from 3-80 million since 1980.
- Up to 44% of CT exams not justified in USA [13].
- The low level of knowledge of dose; only 1:3 doctors received formal training in radiation protection [14].
- The per capit annual effective collective dose from medical imaging varies considerably between countries (Fig.2).

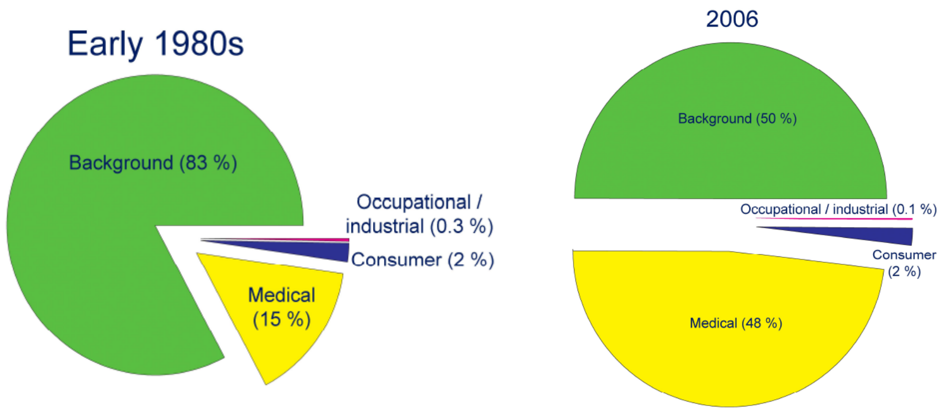

Fig. 1. Components of background radiation in USA in 1980 and 2006. From NCRP 160 [12].

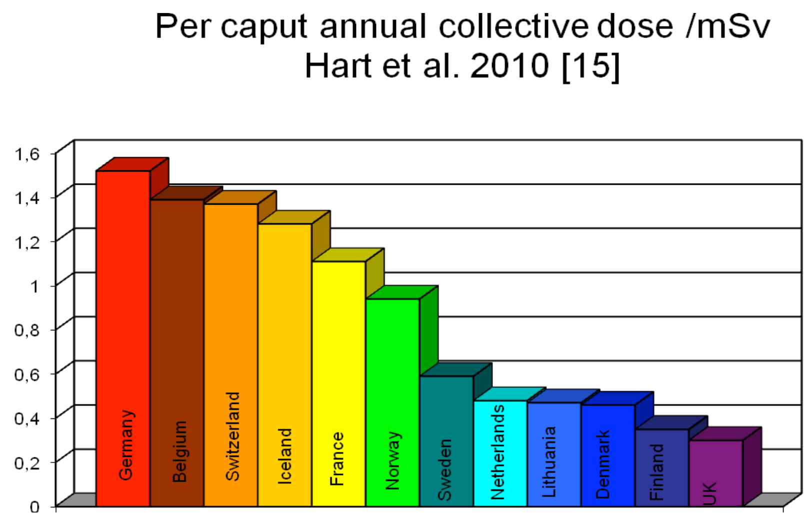

Fig 2. The variation in per capit collective radiation dose in Europe. From Hart et al, 2010 [15].

- Reasons for wasteful diagnostic radiology [2]:
    1. Unnecessary, unhelpful repeated procedures
    2. Investigations whose results are unlikely to affect patient management
    3. Investigating too early in the course of self-limiting conditions
    4. Inappropriate procedure performed which may not fully answer the clinical question, when an alternative investigation may have been best, and
    5. Inadequate clinical information on which to select the best procedure.

Appropriateness: Examples of Referral Guidelines in Europe

Imaging Referral Guidelines have been developed, adopted and adapted by several European Member States and by the European Commission (Figs. 4-7). An integral part of such guidance has been radiation dose information for imaging procedures which is essential to guide the referring physician and patient and to inform advice on radiation risk (Figs. 8-9). Some Guidelines have separate sections dealing with imaging of children.

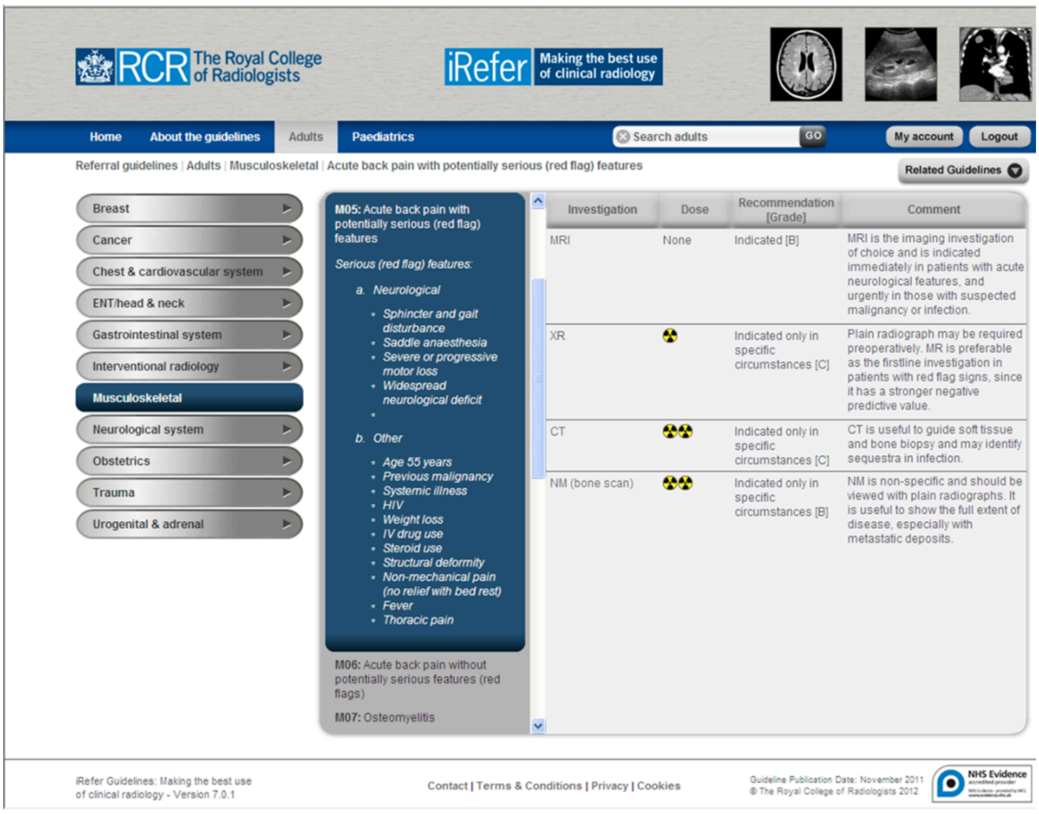

Fig. 4. RCR, iRefer: Making the best use of clinical radiology [2]

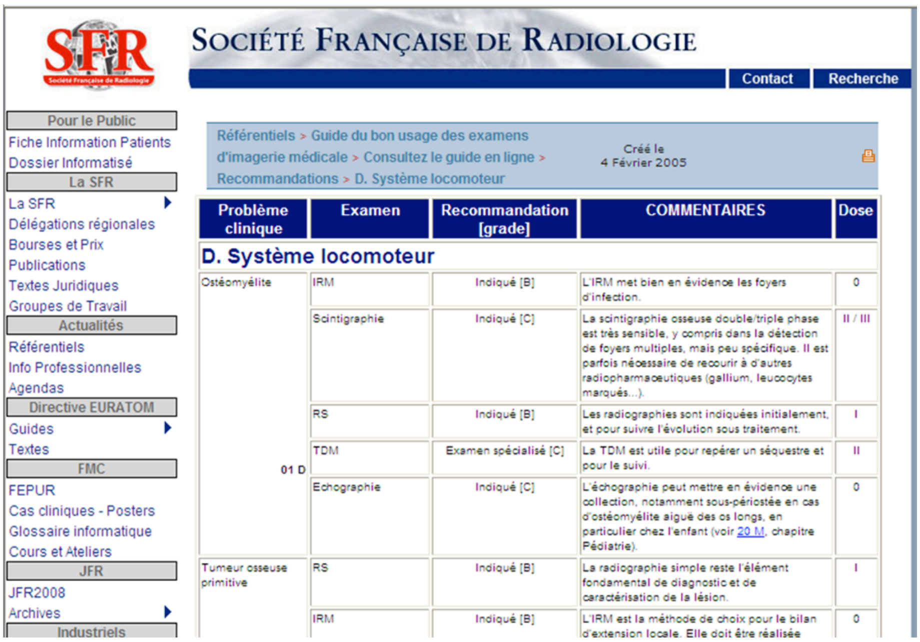

Fig. 5. SFR, Guide du bon usage des examens d'imagerie médicale [4].

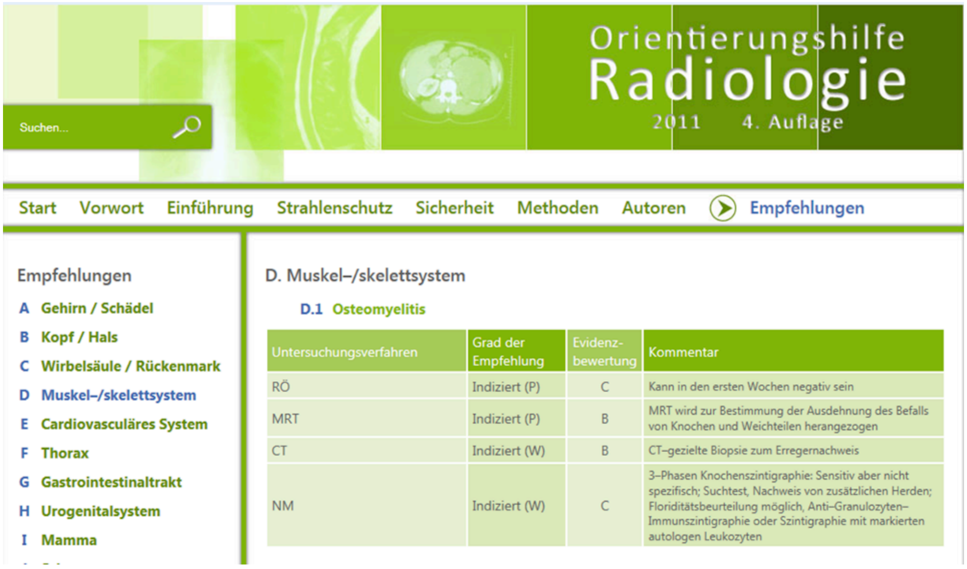

Fig 6. Radiology Guidelines from the Austrian Society of Radiology [16].

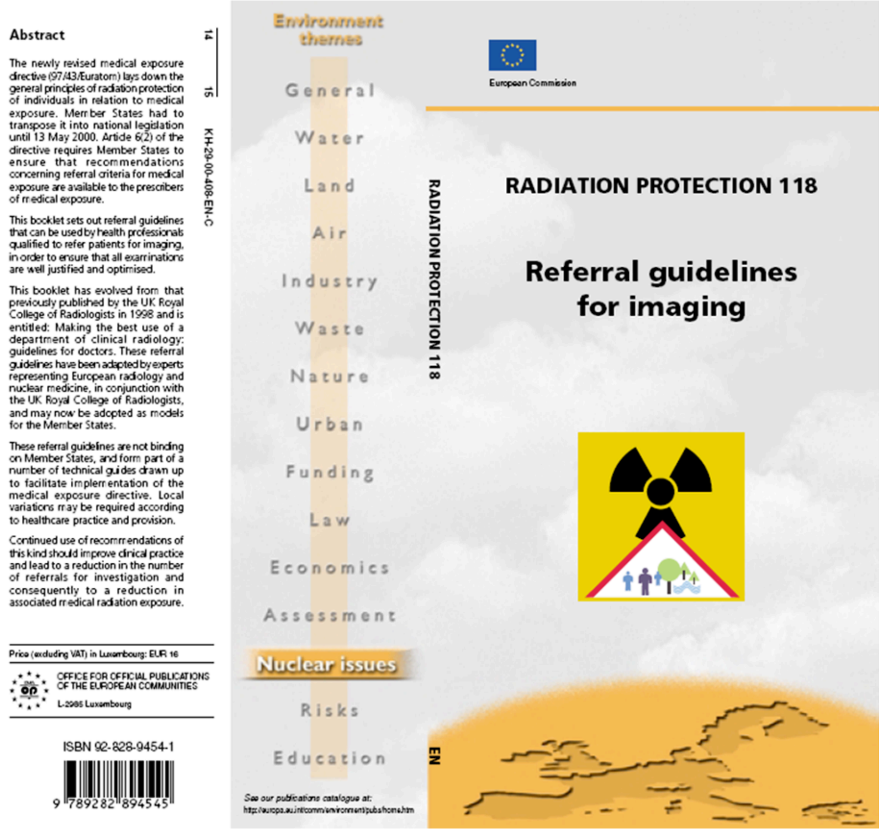

Fig. 7. The European Commission Radiation Protection Referral Guidelines for imaging [3].

Diagnostic procedure	Typical effective dose (mSv)	Equivalent number of chest X-rays	Approx equivalent period of natural background radiation*
**Radiographic examinations**			
Limbs and joints (except hip)	<0.01	<1	<2 days
Chest (single PA)	0.015	1	2.5 days
Skull	0.07	5	12 days
Thoracic spine	0.4	30	2 months
Lumbar spine	0.6	40	3 months
Mammography (2 view)	0.5	35	3 months
Pelvis	0.3	20	1.5 months
Abdomen	0.4	30	2 months
IVU	2.1	140	11.5 months
Barium swallow	1.5	100	8 months
Barium meal	2	130	11 months

Fig. 8. Dose information from the RCR guidelines, iRefer: Making the best use of clinical radiology [2].

Table 3. Band classification of the typical doses of ionising radiation from common imaging procedures[23,26]

Symbol	Typical effective dose (mSv)*	Examples	Lifetime additional risk of fatal cancer/exam
None	0	US;<br>MRI	0
☢	<1	CXR; XR limb, pelvis, lumbar spine; mammography	<1:20,000
☢☢	1–5	IVU; NM (eg, bone);<br>CT head and neck	1: 20,000–1:4,000
☢☢☢	5.1–10	CT chest or abdomen; NM (eg, cardiac)	1: 4,000–1: 2,000
☢☢☢☢	>10	Extensive CT studies, some NM studies (eg, some PET-CT)	> 1: 2,000

*The average annual background dose in most parts of Europe falls within the 1–5 mSv range (☢☢). Cancer risks from radiation vary considerably with age and sex, with higher risks in infants and females.[24] Cancer risk indicated in this table is averaged for adults. This should be taken in the context of the considerably higher 1 in 3 average lifetime risk for cancer and must be balanced against the benefit of the investigation.

Fig. 9. Risk information from the RCR guidelines, iRefer: Making the best use of clinical radiology [2].

Audit: Monitoring and quality improvement of safe radiological practice

Even where available, imaging referral guidelines have not been used as widely as anticipated. Encouragement for use will require greater awareness and education. The need to monitor and reinforce referral guideline use is essential to promote good practice. Initiatives to monitor and promote safe radiological practice include clinical audit, both external [17] (Fig. 10) and local internal audit [18]; voluntary reporting and inspection.

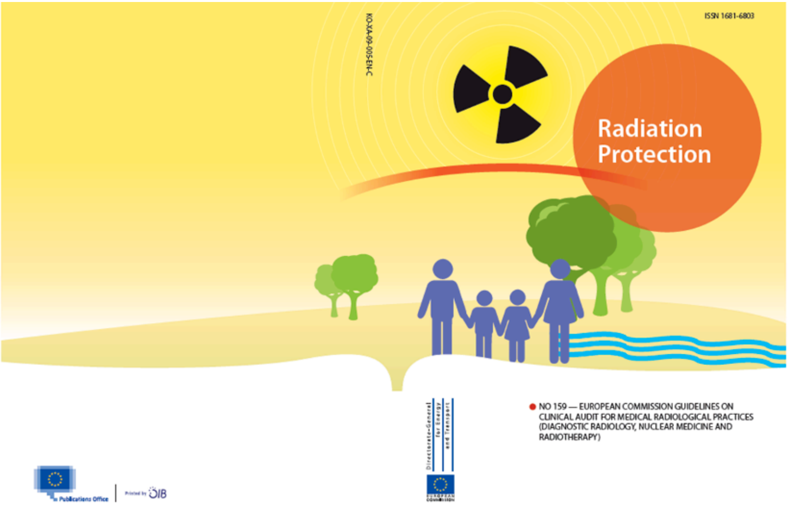

Fig. 10. The European Commission Guidelines on Clinical Audit for Medical Radiological Practices (diagnostic radiology, nuclear medicine and radiotherapy) [17].

Summary

With imaging referral guidelines, the needs, methods for development and value have been established. Good practices are found in Europe (and abroad) but uniform availability, uptake and implementation may not yet be established.

The objectives of the EC Guidelines Project will help address some of these issues. They include:

- A survey of availability of referral guidelines in EU member states and countries taking EC legislation;
- The workshop where the survey findings are disseminated, ideas for European initiatives discussed and the way forward debated;
- Recommendations for future Community action; and
- The final report to the EC within 15 months from project kick-off, a tight time scale but essential for guiding urgent action.

References

1. European Commission. Council Directive 97/43/Euratom on health protection of individuals against the dangers of ionizing radiation in relation to medical exposure. 1997. http://ec.europa.eu/energy/nuclear/radioprotection/doc/legislation/9743_en.pdf
2. Royal College of Radiologists. iRefer: Making the best use of clinical radiology, 7th edition. London: The Royal College of Radiologists, 2012. http://www.irefer.org.uk/
3. [EC RP118] European Commission (2000). Referral Guidelines for Imaging. 2000. http://ec.europa.eu/energy/nuclear/radioprotection/publication/doc/118_en.pdf http://ec.europa.eu/energy/nuclear/radioprotection/publication/doc/118_en.pdf
4. Société Française de Radiologie. Guide du bon usage des examens d'imagerie médicale. http://beclere.sfrnet.org/sitewebpub.nsf http://beclere.sfrnet.org/SFR/Guide_du_bon_usage/player.html
5. International Commission on Radiological Protection, ICRP 103. The 2007 Recommendations of the International Commission on Radiological Protection. ICRP

Publication 103. Annals of the ICRP, 2007. 37(2-4): p. 1 - 332. http://www.icrp.org/docs/ICRP_Publication_103-Annals_of_the_ICRP_37(2-4)-Free_extract.pdf
6. Oakeshott P, Kerry SM, Williams JE. Randomized controlled trial of the effect of the Royal College of Radiologists' guidelines on general practitioners' referrals for radiographic examination. Br J Gen Pract. 1994 Sep;44(386):427-8. http://www.pubmedcentral.nih.gov/picrender.fcgi?artid=1238864&blobtype=pdf
7. Royal College of Radiologists Working Party. Influence of Royal College of Radiologists' guidelines on referral from general practice. BMJ. 1993 Jan 9;306(6870):110-1. http://www.ncbi.nlm.nih.gov/pubmed/8435606?ordinalpos=1&itool=EntrezSystem2.PEntrez.Pubmed.Pubmed_ResultsPanel.Pubmed_DiscoveryPanel.Pubmed_Discovery_RA&linkpos=3&log$=relatedarticles&logdbfrom=pubmed
8. WHO. Global Initiative on Radiation Safety in Healthcare Settings. Technical Meeting Report. 2008. http://www.who.int/ionizing_radiation/about/GI_TM_Report_2008_Dec.pdf
9. WHO. Medical imaging specialists call for global referral guidelines. 2010. http://www.who.int/ionizing_radiation/medical_exposure/referral_guidelines.pdf
10. IAEA and EC. Justification of Medical Exposure in Diagnostic Imaging. 2011. http://www-pub.iaea.org/MTCD/Publications/PDF/Pub1532_web.pdf
11. Malone J, Guleria R, Craven C, Horton P, Järvinen H, Mayo J, O'reilly G, Picano E, Remedios D, Le Heron J, Rehani M, Holmberg O, Czarwinski R. Justification of diagnostic medical exposures: some practical issues. Report of an International Atomic Energy Agency Consultation. Br J Radiol. 2012 May;85(1013):523-38. Epub 2011 Feb 22. http://www.ncbi.nlm.nih.gov/pubmed/21343316
12. NCRP Report No. 160, Ionizing Radiation Exposure of the Population of the United States. http://www.ncrponline.org/Publications/Press_Releases/160press.html
13. Hadley JL, Agola J, Wong P. AJR 2006; 186: 937-942. http://www.ncbi.nlm.nih.gov/pubmed/16554560
14. Soye & Paterson. A survey of awareness of radiation dose among health professionals in Northern Ireland. Br. J. Radiol. 81 (2008),725-729. http://bjr.birjournals.org/cgi/content/abstract/81/969/725
15. Hart et al. Frequency and Collective Dose for Medical and Dental X-ray Examinations in the UK, 2008 www.hpa.org.uk/webc/HPAwebFile/HPAweb_C/1287148001641
16. Bundesfachgruppe Radiologie der Österreichischen Ärztekammer und der Österreichischen Röntgengesellschaft. Orientierungshilfe Radiologie. http://orientierungshilfe.vbdo.at/
17. EC guidelines on clinical audit for medical radiological practice. http://ec.europa.eu/energy/nuclear/radiation_protection/doc/publication/159.pdf
18. RCR. AuditLive 100+ Recipes. http://www.rcr.ac.uk/audittemplate.aspx?PageID=1020&keywordID=47

Talk 2: Referral Guidelines for Imaging in Euratom: Legal Framework and Implementation Efforts

Georgi Simeonov, European Commission

Georgi Simeonov, Directorate-General for Energy, European Commission, Brussels, Belgium
Georgi.simeonov@ec.europa.eu

Legal Basis

The obligation of the EU Member States "to ensure that recommendations concerning referral criteria for medical exposures, including radiation doses, are available to the prescriber of medical exposures" is established by Article 6.2 of Council Directive 97/43/EURATOM (Medical Exposures Directive, MED) [1].

European Referral Guidelines for Imaging

European referral guidelines for imaging were first published by the European Commission, as "Radiation Protection 118" (RP118), in 2000 and further updated in 2003. They were based on the current editions of the Royal College of Radiologists guidelines and adapted by experts representing European radiology and nuclear medicine. RP118 was freely available online and served as a basis for national guidelines published in several European countries.

In 2009 the EC published a tender to update Radiation Protection 118, which failed mostly due to the escalating costs for this work. Later, in September 2010, RP118 was officially withdrawn from publication as "outdated" [2].

Referral Guidelines Project, 2011-2012

In 2011 the European Commission published a new call for tenders [3] with the overall objective to review the situation in EU Member States regarding the fulfilment of their obligations under MED Article 6.2. The specific tasks defined in the tender include:

- The conduction of an EU-wide study on the availability, development and implementation of referral guidelines for radiological imaging in the EU Member States.
- The organisation of a European Workshop with relevant representatives from the EU Member States.
- The development of conclusions of the workshop regarding the need for national and/or Community action.

The contract for this tender was signed at the end of 2011 with a Consortium consisting of:

- ESR European Society of Radiology - Project Coordinator
- RCR Royal College of Radiologists
- SFR Société Française de Radiologie
- CIRSE Cardiovascular and Interventional Radiology Society of Europe
- ESPR European Society of Paediatric Radiology

Observers included the World Health Organizations (WHO), and the International Atomic Energy Agency (IAEA).

As part of the project, a questionnaire was distributed at the end of March 2012 to three contact points in 30 different countries, throughout the EU and also Switzerland, Croatia and Norway. The questionnaire asked the participants not only about the actual status of imaging referral guidelines in the respective country but also for their views on several issues, including on the need of European solutions.

The Directorate General (DG) Energy Perspective

According to the Referral guidelines 118: "Continued use … can lead to a reduction in the number of referrals and also to a reduction in medical radiation exposure. However, the primary objective of the guidelines is to improve clinical practice." In response to this, the DG Energy believes that the development process involves mostly search and grading of clinical evidence, while contribution of radiation protection, e.g. dose per examination [4], is quite limited.

Other potential ways to improve justification include:

- The empowerment of radiologists to refuse / modify examination requests, e.g. through the EC proposal for substituting 'prescriber' with 'referrer' in the revised Euratom BSS [5]
- Provision of education and training to referrers and radiological practitioners, e.g. through European educational initiatives [6]
- Provision of information to patients, also emphasized in the revised Euratom BSS
- Clinical audit [7]

Further European needs could include the continued development of referral guidelines, although national and international work in this area is already occurring. More importantly, the implementation of existing referral guidelines has to be decisively improved, but exactly how has yet to be confirmed, including through work within the current project. The advancement in this area would bring multiple benefits going beyond the narrow area of radiation protection. In order to achieve this contributions would be required from different disciplines. The work should be undertaken within the most appropriate European policy area(s), including health research, electronic healthcare (eHealth) and public health.

References

1. European Commission. Council Directive 97/43/Euratom on health protection of individuals against the dangers of ionizing radiation in relation to medical exposure. http://ec.europa.eu/energy/nuclear/radioprotection/doc/legislation/9743_en.pdf
2. http://ec.europa.eu/energy/nuclear/radiation_protection/publications_en.htm
3. http://ted.europa.eu/udl?uri=TED:NOTICE:130831-2011:TEXT:EN:HTML
4. http://ddmed.eu/
5. European Commission (2012). Proposal for a Council Directive laying down basic safety standards for protection against the dangers arising from exposure to ionising radiation. http://ec.europa.eu/energy/nuclear/radiation_protection/doc/2012_com_242.pdf.
6. http://www.medrapet.eu/
7. European Commission (2009) Radiation Protection 159. European Guidelines on Clinical Audit for Medical Radiological Practices. http://ec.europa.eu/energy/nuclear/radiation_protection/doc/publication/159.pdf

---

Talk 3. Referral Guidelines: the WHO perspective

Maria Perez, The World Health Organization (WHO)

Dr. Maria del Rosario Pérez
Public Health and Environment, World Health Organization, Switzerland
perezm@who.int

Appropriateness is more than a radiation protection issue

The increasing use of radiation in medical imaging and the need to control radiation exposure and minimize health risks, while maximizing the associated health benefits, represent a major challenge. However the appropriateness of usage is not only an issue for radiation in health care. The World Health Organization (WHO) estimated in 2010 that more than half of all medicines are inappropriately prescribed, dispensed or sold. This incorrect use may take the form of overuse, underuse and misuse of prescription or non-prescription medicines.

Good medical practice encompasses radiation safety

For an individual patient, the risk-benefit balance favours the benefit when the procedure is appropriately prescribed and properly performed. The balance is no longer in the patient's favour if the procedure is prescribed without a clear clinical indication or when patients receive a higher dose than is necessary (for example if adult settings are used for children).

The new International Basic Safety Standards (BSS) adopted by the WHO in May 2012 particularly address justification and explicitly mention referral guidelines.
There are three levels of justification outlined:

1. General/overarching justification of the use of ionizing radiation in medicine;
2. Justification of a radiological medical procedure for a given clinical condition; and
3. Justification of a radiological medical procedure for an individual patient.

These justification levels are then further developed in chapter 3 of the BSS, addressing questions such as "what" and "who"?

Need for clinical guidelines

Evidence-based medicine means integrating (i) the best available external clinical evidence from systematic research with (ii) the individual clinical expertise, to consider what may be applicable to or appropriate for an individual patient. It is unrealistic to expect clinicians to keep abreast of all the medical advances reported in primary journals. Instead referral guidelines represent decision-support tools systematically developed to assist practitioners on decision about appropriate healthcare for specific circumstances.

However there can be barriers to guideline implementation. There are many gaps between best available evidence and current clinical practice (evidence-practice gaps). There is a need to identify the barriers to guidelines implementation, to change the practice and close the gaps. In 1996, Pathman et al. developed a four-step model to look at the utilisation of clinical guidelines: awareness, agreement, adoption, adherence. Patterns of "leakage" indicated that it was progressive through the 4 steps.

Potential solutions to these barriers include end-user involvement, local adaptation (format, media), Computerised Physician Ordered Entry (CPOE) systems, to integrate referral guidelines into the daily workflow, education and training.

One of the greatest challenges is to build a global platform to share evidence and resources for guidelines development and update. In collaboration with professional societies and relevant international organizations including the European Commission (EC), the WHO is conducting a Global Initiative on Radiation Safety in Health Care Settings (GI) to mobilise the health sector towards safer use of radiation in medicine. This GI provides multiple opportunities for cooperation with European countries on topics related to radiation safety in medical exposures, with the ultimate goal of promoting appropriate use of radiation in healthcare.

From a public health perspective, the impact of EU developments on radiation protection in healthcare goes beyond the region. The WHO welcomes this EC study on Imaging Referral Guidelines that provides a new opportunity to expand the horizons of EU achievements to a global dimension: jointly building a global platform to share evidence and resources for guidelines development and implementation.

Talk 4: **Referral Guidelines: The IAEA expert's perspective**
Madan Rehani, International Atomic Energy Agency (IAEA)

Madan Rehani, PhD, Radiation Protection of Patients Unit,
International Atomic Energy Agency, Vienna, Austria
madan.rehani@gmail.com, m.rehani@iaea.org

International Atomic Energy Agency (IAEA) has not only been active in optimization action in patient protection but also in appropriateness of imaging to reduce unnecessary exposures of patients. The RPOP website of the IAEA (http://rpop.iaea.org) provides information directed at referring physicians about appropriateness and there is training material for different professionals [1]. In particular there is information on: what is justification and what is the framework; is the referring medical practitioner responsible for justification of radiological procedures?; how should justification be practiced and what knowledge is required for proper justification of a radiological procedure?; is the acquisition of patients' consent important?; when is an investigation useful and what are the reasons that cause unnecessary use of radiation?; what are the reasons for over-investigating; is there any guidance available and the response provides listing of appropriateness criteria and referral guidelines from different sources. The IAEA also recommended triple A (AAA) approach implying appropriateness, awareness and audit [2, 3]. A number of other actions and project results are available as described below.

Requirements in Standards

There are requirements in Basic Safety Standards (BSS) on referral criteria. The international BSS published by the International Atomic Energy Agency (IAEA) in cooperation with a number of organizations states [4] "The specific objectives of the exposure, the clinical circumstances and the characteristics of the individual involved have to be taken into

account through referral criteria developed by professional bodies and the health authority". It further states that the "relevant national or international guidelines shall be taken into account for justification of the medical exposure of an individual patient in radiological procedure". The European BSS, which is at advanced stage of approval [5] states that "Member states shall ensure that referral guidelines for medical imaging, taking into account the radiation doses, are available to the referrers".

Further, the international BSS states under requirement 36, "Registrants and licensees shall ensure that no person incurs a medical exposure unless there has been an appropriate referral, responsibility has been assumed for ensuring protection and safety, and the person subject to exposure has been informed as appropriate of the expected benefits and risks.

In a way, while European BSS emphasizes on availability, the IAEA BSS goes a step in requiring use and appropriate referral. Thus there is emphasis on process and ensuring appropriateness.

IAEA Studies in 40 countries

The IAEA conducted a survey of practice of appropriateness in pediatric CT in 40 less resourced countries and the results have been published [6]. The countries were: Algeria, Armenia, Belarus, Bosnia and Herzegovina, Brazil, Bulgaria, China, Costa Rica, Croatia, Czech Republic, Estonia, Indonesia, Iran, Israel, Kuwait, Lebanon, Lithuania, Malaysia, Malta, Mexico, Montenegro, Moldova, Myanmar, Oman, Pakistan, Paraguay, Peru, Poland, Qatar, Serbia, Singapore, Slovakia, Slovenia, Sri Lanka, Sudan, Syria, Tanzania, Thailand, the Former Yugoslav Republic (FYR) of Macedonia, and United Arab Emirates (UAE).

Also a survey was conducted by the IAEA among 728 referring physicians from 28 countries, which indicated preference for mandatory provisions for justification of a CT examination [7].

In an IAEA study, it was shown that although regulations in many countries assign radiologists with the main responsibility of deciding whether a radiological examination should be performed, in fact radiologists alone were responsible for only 6.3% of situations. Written referral guidelines for imaging were not available in almost half of the CT facilities. Appropriateness criteria for CT examinations in children did not always follow guidelines set by agencies, in particular for patients with accidental head trauma, infants with congenital torticollis, children with possible ventriculo-peritoneal shunt malfunction and young children (<5 years old) with acute sinusitis.

The question, "Who decides whether a CT examination of a pediatric patient is to be performed?" was answered by 127 radiologists. Of these, 72 (56.7%) stated that the decision is usually shared by the radiologist and referring clinician, 47 (37%) said that it is made by the referring clinician, and only eight (6.4%) declared that the decision is made by the radiologist. From a total of 132 answers to another question, "Are written referral guidelines for imaging available in your hospital?" 66 (50%) said "Yes," 59 (44.7%) said "No," and seven (5.3%) answered, "Don't know."

On the question “Is head CT mandatory for a pediatric patient with an accidental head trauma?” the answers are reflective of current practice in the different institutions worldwide. The implementation of clinical practice guidelines to refer pediatric patients with minor head trauma for CT may focus resources on those most likely to benefit from a CT, or may even reduce its usage in pediatric patients [8, 9].

The European Commission’s Referral guidelines for imaging [10] do not recommend CT in patients with a low risk of intracranial injury. CT is recommended in patients with medium to high risk for intracranial injury. According to the NICE clinical guideline 56 [11], CT is recommended in children presenting with loss of consciousness, amnesia, drowsiness, three or more episodes of vomiting, clinical suspicion of non-accidental injury, post-traumatic seizures, Glasgow Coma Scale (GCS) less than 15 for a baby under 1 year old, suspicion of open or depressed skull injury or tense fontanelle, any sign of basal skull fracture, focal neurological deficit, bruise or swelling more than 5cm for a baby under 1 year old and dangerous mechanism of injury.

In response to the first choice examination for an “infant with congenital torticollis”, the large variation in responses amongst the participants was observed to reflect institutional preference in narrowing the differential diagnosis. The European Commission’s Referral guidelines for imaging [10] do not recommend X-ray because the deformity is usually due to spasm with no significant bone changes. If persistent, further imaging (e.g. CT) may be indicated following consultation.

For a “child with clinically suspected appendicitis”, 90% chose US as the first choice examination. This is in line with the ACR appropriateness criteria recommending US as the first investigation. CT may be performed following a negative or equivocal US [12].

For a “child with pleural effusion”, plain X-ray was the initial investigation in 53% and US in 41% of cases. The European Commission’s Referral guidelines for imaging recommend chest X-ray [10]. US may be performed to prove fluid consistency and to guide aspiration.

The first choice examination for a “child with persistent headache” was MRI 48% and CT 45%. The European Commission’s Referral guidelines for imaging [10] recommend MRI in preference to CT because of the absence of ionizing radiation. The ACR appropriateness criteria [12] indicate that MRI and CT are usually not appropriate in children with headache unaccompanied by neurological signs and symptoms.

For a “child with possible ventriculo-peritoneal shunt malfunction”, 63% of radiologists chose CT as the first choice investigation. The current recommendation in the European Commission’s Referral guidelines for imaging [10] is US if practical or MRI in older children. CT may be performed if MRI is unavailable. If CT is used, the CT technique may be modified to reduce the radiation dose because ventricular size is the main object of the CT and noisier images can be tolerated.

For a “small child (< 5years old) with sinusitis”, X-ray was chosen in 72% and CT in 18% of cases. The European Commission’s Referral guidelines for imaging [10] do not recommend imaging for children less than 5 years of age because the sinuses are poorly developed at this age.

Appropriateness criteria for referral for CT examinations in children varied between different countries and did not always follow guidelines set by agencies. Knowledge and use of these guidelines would improve clinical practice and lead to a reduction in the number of unnecessary CT examinations in children.

References

1. Radiation protection of patients website of the IAEA http://rpop.iaea.org
2. Hendee W, Mettler M Jr, Walsh M, Guleria R, Craven C, Sia S, Durand-Zaleski I, Sheehan M, Czarwinski R, Rehani M, Le Heron J, Boal T, Zaknun J; International Atomic Energy Agency (IAEA). Report of a consultation on justification of patient exposures in medical imaging. Radiat Prot Dosimetry. 2009;135(2):137-144
3. Malone J, Guleria R, Craven C, Horton P, Järvinen H, Mayo J, O'reilly G, Picano E, Remedios D, Leheron J, Rehani M, Holmberg O, Czarwinski R. Justification of diagnostic medical exposures, some practical issues: report of an International Atomic Energy Agency Consultation. Br J Radiol. 2011. http://www.ncbi.nlm.nih.gov/pubmed/21343316
4. International Atomic Energy Agency (IAEA) 2011 International Basic Safety Standards for protecting people and the environment. Radiation Protection and Safety of Radiation Sources: International Basic Safety Standards. General Safety Requirements Part 3. No. GSR Part3 (Interim), IAEA, Vienna, Austria
5. European Commission. Proposal for a COUNCIL DIRECTIVE laying down basic safety standards for protection against the dangers arising from exposure to ionising radiation. Draft presented under Article 31 Euratom Treaty for the opinion of the European Economic and Social Committee. http://ec.europa.eu/energy/nuclear/radiation_protection/doc/com_2011_0593.pdf
6. Vassileva J, Rehani MM, Al-Dhuhli H, Huda M. Al-Naemi, Al-Suwaidi JS, Appelgate K, Arandjic D, Bashier EHO, Beganovic A, Benavente T, Bieganski T, Dias S, El-Nachef L, Faj, Gamarra-Sánchez ME, Garcia-Aguilar J, Gbelcová L, Gershan V, Gershkevitsh E, Gruppetta E, Hustuc A, Ivanovic S, Jauhari A, Kharita MH, Kharuzhyk S, Khelassi-Toutaoui N, Khosravi HR, Khoury H, Kostova-Lefterova D, Kralik I, Liu L, Mazuoliene J, Mora P, Muhogora W, Muthuvelu P, Novak L, Pallewatte AS, Shaaban M, Shelly E, Stepanyan K, Teo ELHJ, Thelsy N, Visrutaratna P, Zaman A, Zontar D. IAEA survey of pediatric CT practice in 40 countries in Asia, Europe, Latin America, and Africa: Frequency and Appropriateness AJR Am J Roentgenol. 2012 May;198(5):1021-1031.http://www.ncbi.nlm.nih.gov/pubmed/22528891
7. Rehani MM, Berris T. International Atomic Energy Agency study with referring physicians on patient radiation exposure and its tracking: a prospective survey using a web-based questionnaire. BMJ Open. 2012 Sep 20;2(5). doi:pii: e001425. 10.1136/bmjopen-2012-001425. http://bmjopen.bmj.com/content/2/5/e001425.long
8. Osmond MH, Klassen TP, Wells GA, et al. CATCH: a clinical decision rule for the use of computed tomography in children with minor head injury. CMAJ 2010; 182(4): 341 8.
9. Palchak MJ, Holmes JF, Vance CW, et al. A decision rule for identifying children at low risk for brain injuries after blunt head trauma. Ann Emerg Med. 2003; 42(4): 492 506.
10. European Commission. Referral guidelines for imaging (update 2008). http://ec.europa.eu/energy/nuclear/radioprotection/publication/doc/118_update_en.pdf (accessed 1 May 2011)

11. National Institute for Health and Clinical Excellence. NICE clinical guideline 56. Head injury: triage, assessment, investigation and early management of head injury in infants, children and adults. http://www.nice.org.uk/ (accessed 1 May 2011)
12. American College of Radiology. ACR Appropriateness Criteria. http://www.acr.org/SecondaryMainMenuCategories/quality_safety/app_criteria.aspx (accessed 1 May 2011)

Talk 5: Imaging guidance in Europe: the ESR vision
Guy Frija, European Society of Radiology (ESR)

Prof. Guy Frija, ESR President
guy.frija@egp.aphp.fr

Actions of the European Society of Radiology (ESR) in the field of patient radioprotection include:

- an active participation in EC tenders (EMAN and MEDRAPET);
- an ongoing approach on how to develop Clinical Decision Support Systems in Europe with several Directorate Generals (Energy, Sanco, Connect and Research);
- the methodological development of Clinical Audit in Radiology;
- an international involvement by participating to several meetings organised by IAEA and WHO;
- a contribution to a worldwide approach in Quality and Safety (including Patient Radioprotection aspects) by organising a global summit with the American College of Radiology and the International Society of radiology; and
- the issuing of several papers dealing with patients radioprotection.

In addition the ESR training charter includes a section on radiation protection, and training for undergraduate education, which is in preparation.

The ESR recently welcomed the availability of two recently updated referral guidelines in Europe (France and UK), which in our opinion, might be used as such by the member states, without any need of a supplementary work; however there are pending copyrights issues which have to be solved. Nevertheless, the actual use of Referral Guidelines was strongly questioned in a separate survey made by the ESR towards the national Societies of the MS, Belgium, France, Hungary, Italy, Germany, The Netherlands, Spain, and Ireland. Responses to this survey stated that whilst referral guidelines were available, they were not used in practice. We strongly believe that a comprehensive survey conducted by the consortium on this specific point has to be undertaken.

The different methods used in the field of medicine which have the goal of improving clinical practice by increasing the use of evidence based data, were quickly reviewed, including consensus conferences, meetings, educational sessions, and incentives based policies (P4P). This review showed that the different methods had very limited efficacy (except for P4P approaches which need to be evaluated in the field of imaging). In this context, the first experiences conducted in the US with Clinical Decision Support are looking very promising, and it is our strong belief that this kind of approach must be developed in the EU, with an appropriate level of funding support from the EC.

Talk 6: Referral Guidelines in the UK

Pete Cavanagh, The Royal College of Radiologists (RCR)

Dr. Peter Cavanagh, Dean of the Faculty of Clinical Radiology, (Vice-President, Clinical Radiology), the Royal College of Radiologists, UK
Peter.Cavanagh@southwest.nhs.uk

The Royal College of Radiologists (RCR) has produced the referral guidelines, *iRefer: Making the best use of clinical radiology*,[1] (formerly known as *Making the best use of clinical radiology services*) to help clinicians, radiologists, radiographers and other healthcare professionals to determine the most appropriate imaging investigation(s) for a wide range of clinical problems. Despite the new name, this is the seventh edition of the radiology referral guidelines, demonstrating through more than 20 years' support, the RCR's commitment to the importance of guidelines in the delivery of radiology services for patients. Evidence from the increasing use of diagnostic radiation and publications on the percentage of unnecessary test requests indicates that such a resource is essential.

First published in 1989 on a four-yearly cycle, the RCR published the seventh edition at the end of 2011. Each edition is lead by a Guideline Development Lead, with the first being Professor Adrian Dixon and the current lead being Dr Denis Remedios.

The process and methodology for content review and updates has evolved and developed with each edition. The fourth edition of the guidelines underwent an appraisal by the Health Care Evaluation Unit at St George's Hospital Medical School, while the fifth edition of the guidelines was jointly funded by the RCR and the European Commission.

Each version of the guidelines has grown considerably in size, ranging from just 73 guidelines in the first edition to 307 in the seventh.

Content development

The iRefer process recognises that preparation of evidence-based guidelines is a demanding task, and one that requires a rigorous approach to routine practices and the assimilation of new evidence. The design is aimed to minimise geographical and personal bias through a Delphi consensus process and to consult widely to gather appropriate foreign language and grey literature to further strengthen the evidence base. The process has evolved and improved from edition to edition and now involves hundreds of experts (RCR members and Fellows representing 10% of the Clinical Radiology Faculty) covering all modalities and procedures review and update the guidelines, with a nominated Expert Lead for each section. Extensive literature searches are carried out by the Delphi Search Co-ordinator, involving thousands of papers – 100,000 references in raw search, 30,000 references considered, 3,000 used.

Each edition of the guidelines has been subject to external peer review. Wide consultation of the seventh edition of the guidelines was undertaken among more than 100 organisations, including Royal medical Colleges, learned bodies, specialist societies, professional associations and other professional groups in the UK and Europe. To ensure that the patient remains at the centre of this work, our Patients' Liaison Group was involved at every stage of the process.

This seventh edition of imaging referral guidelines marks a significant leap forward in guideline development. The enhanced guidelines methodology has been accredited by NHS Evidence, managed by the National Institute for Health and Clinical Excellence (NICE) in the UK. In this edition of the guidelines, the Delphi process was used for **every** guideline further strengthening the evidence base. There has also been consideration of relevant clinical guidelines to inform the process and inclusion of important clinical (red flag) features where relevant. The seventh edition features an updated Table 2. Typical effective doses from diagnostic medical exposure based on the latest Health Protection Agency (HPA) survey from 2008[2]. The seventh edition also features the following brand new guidelines:

<u>Cancer</u>
CA23 – Colon cancer: diagnosis
CA24 – Colon cancer: staging
CA25 – Colon cancer: follow–up
CA26 – Rectal cancer: diagnosis
CA27 – Rectal cancer: staging
CA28 – Rectal cancer: follow–up
CA29 – Anal cancer: diagnosis
CA30 – Anal cancer: staging
CA31 – Anal cancer: follow–up
CA56 – Melanoma: diagnosis
CA57 – Melanoma: staging
CA58 – Melanoma: follow–up

<u>Gastrointestinal system</u>
G33 – Asymptomatic patients 50–75 years old with a positive faecal occult blood test on screening for bowel cancer

<u>Urogenital & adrenal</u>
U22 – Asymptomatic men with elevated PSA

The Delphi process
The Delphi consensus is used to agree recommendations, comments and grading of evidence for each guideline. These Delphi groups comprise approximately ten experts and a mix of specialty and modality base. Consensus is reached with 75% participation and 75% agreement at 5, 6 or 7 on a 7-point Likert scale. Expert bias is avoided by anonymising data and geographical bias avoided by use of Delphi experts from different centres.

Evidence for each guideline identified through the literature search supplemented by the Delphi group or through consultation is graded by Delphi members and Lead. Evidence levels for diagnostic, therapeutic, prognostic and economic studies were based on the levels of evidence for primary research adapted from *The Journal of Bone and Joint Surgery*.[3]

Who are they for?
Aimed at GPs, referring clinicians, radiologists, radiographers, and other healthcare professionals and healthcare organisations, these guidelines steer referrers clearly through disease and system-based imaging, help with justification, avoid the chief causes of

unnecessary patient irradiation and the wasteful use of radiology, and ensure the best use of imaging for the benefit of patients. Designed to be used in both primary and secondary care, they are particularly helpful for the non-specialist referrer.

The guidelines have international application in the global imaging world, with adoption in Ireland, India, Hungary, Japan, Russia, Portugal, Norway, Netherlands, Canada, Croatia and Saudi Arabia.

Challenges

With so many benefits, the case for guidelines is obvious but despite this the RCR, like others, struggles to get healthcare professionals to embrace their use. It is difficult to get referring clinicians to use them without some form of encouragement, effective feedback or sanctioning. The reasons for this are many, including issues around time pressures, inaccessibility of guidelines, information overload, mixed messages from different guidelines, and patient expectations.

The future

Added to the challenges of implementation is the ongoing production of up-to-date evidence-based guidelines. The RCR has relied heavily on the enthusiasm and dedication of a significant number of its members giving up their time to support this work. Even so, there is a considerable cost required to resource the process and the production of the final versions, particularly as the process becomes, rightly, more robust and the delivery more complex. In the past, this process has been supported by funding nationally outside the College, but recurrent support in its entirety has proved difficult and so the challenge is to look at other models of ensuring effective use of the guidelines.

The RCR is committed to the continued publication of the guidelines and to an ever-improving process of update to ensure that the UK imaging population continues to be the best informed in the world. The RCR is particularly keen to develop decision support software based on its guidelines, so that *iRefer* can be used exactly when needed – when the decision to refer is made.

References

1. The Royal College of Radiologists. *iRefer: Making the best use of clinical radiology* [Internet]. London: The Royal College of Radiologists, 2012. (www.irefer.org.uk)
2. Hart D, Wall BF, Hillier MC, Shrimpton PC. *HPA-CRCE-012 – Frequency and collective dose for medical and dental X-ray examinations in the UK, 2008*. Didcot: HPA, 2010. http://www.hpa.org.uk/web/HPAwebFile/HPAweb_C/1287148001641
3. The Journal of Bone and Joint Surgery instructions to authors. http://www2.ejbjs.org/misc/instrux.shtml

---

Talk 7: Referral guidelines in France

Philippe Grenier, the Société Française de Radiologie (SFR)

Prof. Philippe Grenier, Former General Secretary of the Société Française de Radiologie and Coordinator of the French Referral Guidelines
Philippe.grenier@psl.aphp.fr

In 2003, the European Directive EURATOM 97/43 was transposed in the French law. Despite the European Society of Radiology (ESR) recognized the referral guidelines from the Royal College of Radiology (RCR) and proposed to the national societies to use these guidelines after translation in their national language, the French Society of Radiology [Société Française de Radiologie (SFR)] and the French Society of Nuclear Medicine [Société Française de Médecine Nucléaire (SFMN)] decided to develop French referral guidelines for the clinical use of medical imaging (Guide du Bon Usage des Examens d'Imagerie Médicale).

Two reasons explained this decision. The first was a translation of the European Guidelines in French which was of insufficient quality, and the second was the willingness to initiate appropriation of the guidelines by French professionals.

The main objectives of these referral guidelines were not only to avoid the non-clinically indicated examinations, and to improve the clinical practices by rationale of imaging examination indications, but also to serve as a standard of reference for clinical audits. This initiative was supported by the French national authorities [Haute Autorité de Santé (HAS) and Autorité de Sûreté Nucléaire (ASN)]. It was decided to use the methodology of Delphi consensus for harmonization with international guidelines, following in this way the footprints of the RCR.

A steering committee**,** including representatives of both societies (SFR and SFMN), national authorities [HAS, ASN and Institut de Radioprotection et de Sûreté Nucléaire (IRSN)], and professional organizations (union of private radiologists, union of public hospital radiologists, academic colleges of radiologists and nuclear medicine physicians) was established.

Fourteen subcommittees were created representing the different subsections of medical imaging (neurology, head and neck, musculoskeletal, vascular disease, chest diseases, gastrointestinal, gynecology/obstetrics, breast, paediatrics, endocrinology, lymphoma, cardiology, urogenital, polytrauma). Every subcommittee included three main components: a writing group made of 2 to 9 expert radiologists and nuclear medicine physicians, a multidisciplinary evaluation group made of 12 to 20 experts (50% radiologists and nuclear medicine physicians, and 50% clinicians), and a multidisciplinary reading group made of 30 to 60 experts (50% radiologists and nuclear medicine physicians and 50% clinicians). The expert radiologists were selected from the different subspecialty societies of medical imaging. The expert clinicians were nominated by the scientific societies of clinicians in all specialties in medicine and surgery. Twenty-five of these scientific societies accepted to participate in the process and designated experts.

The writing group of every subcommittee was in charge to establish the first proposals for recommendations. The experts selected items representing various clinical situations (symptoms or suspected disease), analysed the bibliography from the five previous years

after interrogation of international data bases, summarized their analysis and wrote a list of arguments to support their recommendations. Then they proposed recommendations presented in five columns: entry word (clinical symptom or suspected disease), imaging modalities, recommendations of indications, grade of recommendation, comments, and level of radiation effective dose. All imaging modalities and techniques were taken into consideration, not only those delivering ionizing radiation such as radiography, CT, nuclear medicine, angiography but also ultrasound and magnetic resonance imaging. For every clinical situation and for every imaging modality, the level of recommendations included: indicated, indicated only in particular situations (described in the comments column), specialized examination (complex or expensive examination ordered only by experimented physicians in the field of the disease, whose indication needs a multidisciplinary consultation or a consultation with a radiologist subspecialized in the field), not indicated initially (but may be considered according to the patient outcome or specific patient-related conditions), contra-indicated.

The grading of recommendations permitting to express evidence-based diagnostic impact included three levels: Grade A: high level of scientific proof (randomized controlled trials, meta-analysis); Grade B: intermediate level of scientific proof (comparative non randomized studies, cohort studies, randomized comparative studies of mild power); Grade C: low level of scientific proof (www.has-sante.fr). For every modality, the intensity of effective dose was evaluated on the basis of national reference doses provided by the national organization of radioprotection (IRSN). The scores ranged from grade 0 (0 mSv), grade I (< 1mSv), grade II (1-5 mSv), grade III 5-10 mSv, and grade IV (> 10 mSv).

In every subcommittee, the multidisciplinary evaluation group was in charge to analyse the recommendations proposed by the writing group. The guidelines, bibliography analyses, and lists of arguments were sent to the members who were asked to reply to a questionnaire using a scoring scale (1-9) on every component of each item (1: inappropriate, 9: fully appropriate). Medians of scores were calculated. Then, the members met together with the leaders of the writing group at the SFR office. All items having a median score lower than 7 were discussed in order to get to a formalized consensus between experts. The following step consisted to a second evaluation using the same scale and questionnaire, requested either on site at the end of the meeting or in the 48 hours following the meeting. This was considered as the formalized consensus between experts.

The multidisciplinary reading group was in charge to analyse the recommendations proposed by the formalized consensus. The recommendations were sent to the members of this group for reading and comments, and the finalization of the recommendations (taking into account the replies) were made by the subcommittee leaders and the members of the steering committee.

The first edition of the French referral guidelines came out in 2005. It was available on line and published on booklets. The guidelines included 381 clinical situations, and 889 imaging modality recommendations of grade A (n = 62), grade B (n = 618) or grade C (n = 209). Five-hundred and seventy-five physicians were involved in the process including 315 radiologists and nuclear medicine physicians and 260 clinicians. The booklets were sent to all members of the both imaging societies (SFR and SFMN) and all experts having participated to the procedure. Booklets were also sent to the associated scientific societies and national health

authorities. The rest of the booklets were sold on demand by the SFR. An electronic version of the document was put on different websites (SFR, SFMN, HAS and ASN). Thirty per cent of cost payment was supplied by the ASN. The rest was taken in charge by the SFR. Unfortunately there was not a general distribution of the document to the general practitioners and specialists in the country. There was no national publicity for the document provided by national authorities. The electronic version of the guidelines was static (PDF) without software permitting rapid consultation. No assessment of the guidelines impact was performed.

In 2009, it was decided to prepare a new version of the referral guidelines. A new steering committee and new subcommittees were established. The analysis of the bibliography was asked for the previous 10 years. The list of clinical entry words was submitted to a panel of general practitioners and 20% of items were changed compared to the first edition. The same Delphi process (Consensus formalisé entre experts, version longue) as that used in the first edition was applied. This new version is only electronic, run on specific software. It is available on line and can also be uploaded on computers and tablets. An application for smartphones is in course of preparation. The official presentation of this new edition will be done at the “Journées Françaises de Radiologie” in Paris on October 2012. The French authorities of health have promised a large publicity for the guidelines.

The college of academic radiologists [Collège des Enseignants en Radiologie de France (CERF)] has decided to incorporate the referral guidelines in the training programmes of students at medical faculties. A new committee will be in charge to ensure continuous update of the guidelines. These guidelines might be used as a frame of reference in continuous professional development programmes [Développement Professionnel Continu (DPC)] which is the new format for continuous medical education (CME) in France. The SFR has raised the project to integrate the referral guidelines in clinical decisions system support for MR requests.

In summary, this experience of developing referral guidelines at a national level has made it clear that to set up referral guidelines is a long and costly process, involving a high number of physicians. A Delphi process, performed at a national level, certainly helps appropriation of referral guidelines by radiologists and clinicians in the country. Advertisement, distribution, reminders, and education are necessary to ensure the use of guidelines by physicians, and, a web version of guidelines should be recommended to facilitate practical use, availability, distribution, and continuous update.

Société Française de Radiologie (SFR)	www.sfrnet.org
Société Française de Médecine Nucléaire (SFMN)	www.sfmn.org
Autorité de sûreté nucléaire (ASN)	www.asn.fr
Institut de Radioprotection et de Sûreté Nucléaire (IRSN)	www.irsn.fr
French Society of Radiology Referral Guidelines	http://gbu.radiologie.fr

---

Talk 8: Referral Guidelines in Western Australia
Richard Mendelson, Western Australian Health Department

Dr. Richard Mendelson, Western Australian Health Department, Perth, Australia
richard.mendelson@health.wa.gov.au

Diagnostic Imaging Pathways (DIP) is a suite of evidence-based and consensus based guidelines that has been developed over many years to assist clinicians to choose the most appropriate diagnostic examinations according to best practice and evidence. It is only published electronically and is freely available from the intranet within the public hospital system in Western Australia and from the internet: www.imagingpathways.health.wa.gov.au.

The intellectual property of DIP is owned by the Western Australian Health Department. Editorial control and management of DIP is based at Royal Perth Hospital, but Editorial Panel members, contributors and advisors are sourced from other public and private health institutions in WA and, indeed, from interstate and even overseas.

Within the public health system, the aim of DIP is to be a tool for quality control and demand management, through its guidance to clinicians to choose the appropriate imaging in common clinical scenarios.

DIP contains more than 150 pathways, which are relevant to all of the organ systems and common clinical scenarios. Each of the pathways is laid out as a diagnostic flowchart. Commencing with a presenting condition (e.g. low back pain) the user can step through various clinical possibilities (e.g. back pain +/- sciatica) and indications for investigation (e.g. 'red flags'), to receive advice on a sequence of examinations which is recommended according to broad consensus and the best available evidence. Access to supporting information and source references is provided. References are graded according to the Oxford system for levels of evidence. DIP is thought to be unique in its format; rather than the tabular form of recommendations used by other imaging guideline developers DIP uses an algorithmic or flow-chart structure. This allows for a 'layered" format for users to access as much or as little background information as they wish. At the minimum level they can view the flowchart only, but behind the flow charts is accessible, referenced narrative text which forms the basis for the recommendations.

DIP also contains general information about diagnostic imaging, information for patients and carers, image galleries of normal anatomy and pathology, and teaching points for students.

The clinical and academic content is developed, continuously reviewed, and updated at least quarterly under the direction of an Editorial Panel. The panel obtains advice from a large network of 'provider' and 'referrer' contributors which includes imaging specialists with subspecialty interests, medical and surgical specialists, and general practitioners. The recommendations in DIP are therefore evidence and consensus-based.

The application is widely accepted and used and has achieved national and international acclaim. It receives about 7 million 'hits' per year from users worldwide.

DIP is endorsed by the Royal Australian & New Zealand College of Radiologists, is accredited by the Geneva based Health On The Net Foundation, and meets standards for partnership with HealthInsite and the Joanna Briggs Institute. DIP has been accepted as a member of the Guidelines International Network.

The Australian National Health & Medical Research Council (NHMRC) provides access to DIP via its Clinical Practice Guidelines Portal.

Importantly, DIP has achieved accreditation from NICE - National Health Service Evidence (UK). Effectively this means that DIP complies with the AGREE II criteria for development of guidelines.

DIP has been adopted into the curricula of several Australian medical schools including the University of Western Australia and the University of Notre Dame Australia.

The World Health Organisation (WHO) is collaborating with the International Radiology Quality Network (IRQN) on the development of imaging referral guidelines. Draft WHO/IRQN guidelines are based on three existing sets of diagnostic imaging guidelines from around the world, one of which is DIP.

Despite DIP's widespread use, there is evidence from our own studies and those of others that 'stand-alone' guidelines have limited utility in altering requesting behaviour among referring clinicians. Therefore, we are developing and evaluating an electronic request/decision support tool which integrates the academic content of DIP into the requesting process for diagnostic imaging so that imaging recommendations are a seamless part of the clinicians' work flow.

Talk 9: Referral Guidelines in Canada

Martin Reed, The Canadian Association of Radiologists (CAR)

Dr. Martin Reed,
Chair, Guidelines Working Group, Canadian Association of Radiologists
Department of Radiology, Children's Hospital, Winnipeg, Canada
mreed@exchange.hsc.mb.ca

The Canadian Association of Radiologists (CAR) referral guidelines were initially developed as a result of a strategic planning process which was carried out in 2004. The first of three goals identified by that process was "to actively participate in health system cost control through control of inappropriate utilization". In order to achieve this, a guidelines working group was set up. Recognizing that the CAR did not have the resources to develop its own complete set of guidelines a search was undertaken to determine if any sets of radiology referral guidelines were available which we could adopt and adapt. The committee concluded that the Royal College of Radiologists guidelines, *Making the Best Use of the Department of Clinical Radiology: Guidelines for Doctors* (5th Edition) would be the most appropriate for our use. The Royal College of Radiologists kindly gave us permission to use these guidelines. The guideline committee reviewed all the guidelines. Each section was sent out to independent reviewers who were members of the CAR and had expertise in the appropriate specialty area.

The guidelines were also sent to the Canadian Association of Nuclear Medicine. A presentation on the CAR referral guidelines was also made to the National Specialty Societies meeting which was held in conjunction with the annual meeting of the Canadian Medical Association (CMA) in 2005, and the guidelines were circulated to all the specialty societies for comments. The comments received from all these reviewers were all reviewed by the committee, and the guidelines were finalized. For the most part this involved only minor changes to adapt the guidelines to the Canadian context.

The guidelines were published in both English and French versions in booklet form in 2005. However, recognizing that a book of guidelines may not be the best way of ensuring their utilization the guidelines were also made available on the Internet through password protected websites including the CAR website, the CMA Infobase and the website of the College of Family Physicians. A PDF version was also made available on CD. The guidelines were quite widely circulated and the first English printing of one thousand copies was sold out and a second printing had to be made.

The CAR has recently undertaken and completed a revision of its guidelines which are now freely available on the website (http://www.car.ca/en/standards-guidelines/guidelines.aspx). The CAR is committed to continuing to improve and update their referral guidelines, to encourage their use as widely as possible in the physician community in Canada.

Talk 10: Referral Guidelines in the USA
Michael Bettmann, American College of Radiology (ACR)

Michael Bettmann, MD, FACR, FAHA
Chair, ACR Appropriateness Criteria Oversight Committee, Co-Chair, ACR Task Force on Decision Support

Basic concerns in the utilization of imaging include its overuse, inappropriate use, increasing costs and radiation exposure. The American College of Radiology (ACR) Appropriateness Criteria® are based on the best-available clinical data. The intended uses of these criteria include education and clinical decision guidance. In a specific situation, if the healthcare provider is considering an imaging study they should ask the question what study, *IF ANY*, is most likely to provide the necessary information?

Some of the potential reasons for inappropriate imaging include:

- Patient expectations and demands for imaging
- Concerns of liability exposure if diagnosis is delayed
- Conflict of interest presented by physician ownership of imaging equipment (self-referral)
- Lack of specific guidance from Radiologists
- Lack of knowledge by ordering physicians and other providers (increasing number of exams ordered by non-physicians)-e.g., “customary practice”

Table 1: Medical imaging procedures with largest contribution to cumulative effective dose.

Table 3. Medical Imaging Procedures with Largest Contribution to Cumulative Effective Dose.*

Procedure	Average Effective Dose	Annual Effective Dose per Person	Proportion of the Total Effective Dose from All Study Procedures
	millisieverts		%
Myocardial perfusion imaging	15.6†	0.540	22.1
CT of the abdomen	8	0.446	18.3
CT of the pelvis	6	0.297	12.2
CT of the chest	7	0.184	7.5
Diagnostic cardiac catheterization	7	0.113	4.6
Radiography of the lumbar spine	1.5	0.080	3.3
Mammography	0.4	0.076	3.1
CT angiography of the chest (noncoronary)	15	0.075	3.1
Upper gastrointestinal series	6	0.058	2.4
CT of the head or brain	2	0.049	2.0
Percutaneous coronary intervention	15	0.043	1.8
Nuclear bone imaging	6.3	0.035	1.4
Radiograph of the abdomen	0.7	0.028	1.1
CT of the cervical spine	6	0.020	0.8
CT of the lumbar spine	6	0.018	0.7
Chest radiograph	0.02‡	0.016	0.7
Thyroid uptake	1.9	0.016	0.7
Intravenous urography	3	0.014	0.6
CT of the neck	3	0.014	0.6
Cardiac resting ventriculography	7.8	0.014	0.6

* Average effective doses for these imaging procedures are based on data from Mettler et al.[10]
† Calculation of the average radiation dose for myocardial perfusion imaging with the use of single-photon-emission CT relied on dose coefficients from a detailed review of radiation dosimetry of specific cardiac radiopharmaceuticals,[17] median injected radiopharmaceutical doses (millicuries) from the guidelines of the American Society of Nuclear Cardiology,[19] and distributions of the use of various protocols in the United States.[20]
‡ This dose is the effective dose for a posteroanterior study of the chest.

Some of the potential reasons for inappropriate imaging include:

- Patient expectations and demands for imaging
- Concerns of liability exposure if diagnosis is delayed
- Conflict of interest presented by physician ownership of imaging equipment (self-referral)
- Lack of specific guidance from Radiologists
- Lack of knowledge by ordering physicians and other providers (increasing number of exams ordered by non-physicians)-e.g., “customary practice”

<u>Ideal clinical imaging guidelines are:</u>

- Evidence-based
- Produced by experts in imaging and supplemented by others
- Consistent, with a transparent methodology
- Updated regularly (for example every 2 years)
- Widely accepted
- Readily available-on-line, as a database, as part of Computerised Physician Order Entry (CPOE) or Decision Support Systems (DSS)

ACR Appropriateness Criteria® were developed to provide data-based guidance to requesting physicians, radiologists, and radiation oncologists, in making initial decisions about diagnostic imaging and therapeutic techniques.

In conclusion, the ACR Appropriateness Criteria®:

- Provide a sound, transparent, reproducible methodology
- Are data-based, supplemented by expert opinion
- Must “translate” to achieve electronic medical record (EMR)usability
- Demonstrate that sound imaging guidelines, incorporated into EMRs, are necessary
