# Supplementary material for: Imaging referral guidelines in Europe: now and in the future—EC Referral Guidelines Workshop Proceedings
Source: Insights Imaging. 2013 Dec 13;5(1):9–13. doi: 10.1007/s13244-013-0299-8 (PMC3948903; doi:10.1007/s13244-013-0299-8)
Supplement: Supplementary file 2 — Workshop proceedings, Session 2 Specific Issues (PDF 989 kb) [file 13244_2013_299_MOESM2_ESM.pdf]

Project ENER/D4/315-2011
Implementation of Council Directive 97/43/Euratom requirements concerning referral criteria for medical imaging in the European Union

IMAGING REFERRAL GUIDELINES IN EUROPE: NOW AND THE FUTURE. EC REFERRAL GUIDELINES WORKSHOP Billrothhaus, Vienna. September 20-21 2012

Workshop Proceedings

Session 2: Specific Issues

Chair: Mario Bezzi, Cardiovascular and Interventional Radiological Society of Europe (CIRSE)
Rapporteur: Nick Ashford, Royal College of Radiologists (RCR)

Talk 11: Particular paediatric points

Jean-François Chateil, European Society of Paediatric Radiology (ESPR)

Jean-François Chateil MD, PhD
Member of the board of the European Society of Paediatric Radiology, past president
jean-francois.chateil@chu-bordeaux.fr

Imaging referral guidelines in Europe: Specific Aspects in Children

Why is there a need for specific recommendations for children? Children demonstrate a higher sensitivity to ionizing radiations. Pathology can be different in children relative to adults, and different imaging procedures can be carried out. Specific diagnostic reference levels are required, and, in addition, a need for specific guidelines for children has been expressed in a European survey.

Some of the difficulties associated with the establishment of specific recommendations for children include a lack of consensus between paediatricians, radiologists, and countries, heterogeneity of equipment and heterogeneity of formation.

There is a real need to control radiation level with paediatric imaging. The risks associated with medical irradiation are higher at a younger age, particularly the potential carcinogenic effect (see Figure 1.). Organs are particularly sensitive to radiation at younger age, cells are dividing faster, and the expected remaining lifetime is much longer than in older age.

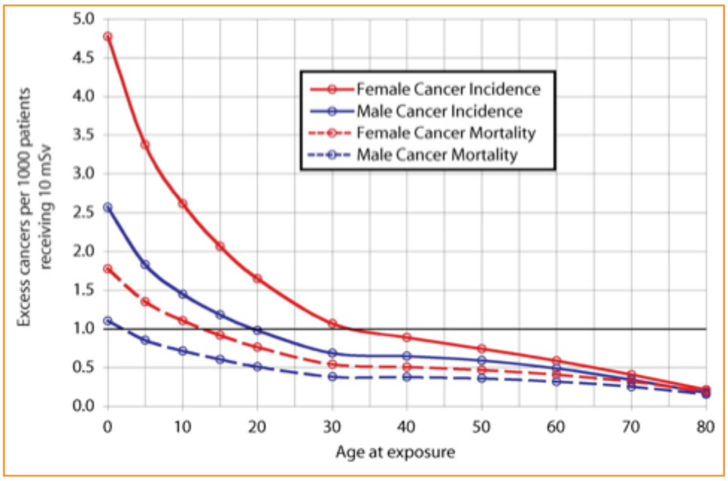

Fig. 1. Additional risk of cancer induction by ionising radiation at different ages.

What is the real risk with paediatric imaging?

In the United States, of approximately 600,000 abdominal and head CT examinations annually performed in children under the age of 15 years, a rough estimate is that 500 of these individuals might ultimately die from cancer attributable to the CT radiation.

Further, a paper was published in the Lancet in June 2012 which showed the relationship between radiation exposure from CT scans in childhood and subsequent risk of leukaemia and brain tumours (Pearce et al. 2012). The paper concluded that Use of CT scans in children to deliver cumulative doses of about 50 mGy might almost triple the risk of leukaemia, and doses of about 60 mGy might triple the risk of brain cancer.

In conclusion:

- Radiation-induced effects are greater in children than they are in adults
- Conventional radiography may represent a low risk, however CT represents a real risk to children, in relation with relative delivered high dose, cumulative effect and longer expected lifetime.
- Some of the educational objectives for paediatric radiology include:
  - Strong justification of explorations with radiation
  - Use of substitutive imaging methods without radiation (US, MRI)
  - ALARA attitude every day: optimization
- Imaging Referral guidance specific for children is essential, with diffusion to general practitioners, paediatricians and radiologists.

References

1. Brenner D, Elliston C, Hall E, Berdon W. Estimated risks of radiation-induced fatal cancer from pediatric CT. AJR Am J Roentgenol. 2001 Feb;176(2):289-96.
2. Brenner DJ, Hall EJ. Computed tomography--an increasing source of radiation exposure. N Engl J Med. 2007 Nov 29;357(22):2277-84.
3. Linet MS, Kim KP, Rajaraman P. Children's exposure to diagnostic medical radiation and cancer risk: epidemiologic and dosimetric considerations. Pediatr Radiol. 2009 Feb;39 Suppl 1:S4-26.
4. Oikarinen H, Merilainen S, Paakko E, Karttunen A, Nieminen MT, Tervonen O. Unjustified CT examinations in young patients. Eur Radiol. 2009 May;19(5):1161-5.

5. Nievelstein RA, van Dam IM, van der Molen AJ. Multidetector CT in children: current concepts and dose reduction strategies. Pediatr Radiol. 2010 Aug;40(8):1324-44.
6. Pearce MS, Salotti JA, Little MP, McHugh K, Lee C, Kim KP, et al. Radiation exposure from CT scans in childhood and subsequent risk of leukaemia and brain tumours: a retrospective cohort study. Lancet. 2012 Jun 6
7. Zanca F, Demeter M, Oyen R, Bosmans H. Excess radiation and organ dose in chest and abdominal CT due to CT acquisition beyond expected anatomical boundaries. European radiology. 2012 Apr;22(4):779-88.

Talk 12: Referral guidelines and Interventional Radiology

Mario Bezzi, Cardiovascular and Interventional Radiological Society of Europe (CIRSE)

Mario Bezzi, MD
Aggregate Professor of Radiology at the University of Rome Sapienza, Italy
Mario.bezzi@uniroma1.it

Image-guided interventional procedures are performed in large numbers in Europe and in the United States and the number of procedures performed annually has increased over the past 20 years (1–3). While the benefits of Interventional Radiology (IR) to patients are extensive and beyond dispute, there are also some procedure-related risks. One of these risks is that many IR procedures have the potential to produce patient radiation doses high enough to cause radiation effects and occupational doses to interventional radiologists high enough to cause concern. Management of radiation exposure is therefore essential for these procedures. This is achieved by education in radiation safety which is part of general radiology training and IR training.

The key issues in radiation exposure management can be summarized in: patient selection, procedure performance, patient monitoring, and appropriate documentation and follow-up. Patient selection is therefore the first key point in a strategy of dose reduction.

The decision to refer a patient to a specialist IR service is a crucial point in his/her management. Despite its importance, however, there has been little research in this area. Many retrospective evaluations of referrals seek to quantify the level of appropriateness, often from one stakeholder’s perspective. Careful analysis reveals that referrals are more complex. They reflect the needs and expectations of individual patients and their families, the knowledge and experience of the individual practitioner, and the range, type and level of services available locally, as it is for Diagnostic Radiology.

There are several differences between Diagnostic Radiology (DR) and IR that we must consider when discussing any proposed Referral Guidelines for IR procedures. DR deals with diagnostic imaging investigations while IR deals with image guided treatments. Imaging investigations are carried out in all primary care centres while IR procedures are most often offered in secondary/tertiary centres. The radiation exposure for most diagnostic imaging tests is standardized, while the same IR procedure may cause different radiation doses in different patients depending on procedural complexity, standard of equipment and body habitus. Importantly, just like a surgical procedure, an IR procedure is a consultation between the Interventional radiologist and the patient where the procedure, risk benefit

ratio and alternatives are discussed and consent obtained. Each consult is an individual transaction and generalizations are difficult to construct.

Concerns over patient radiation doses are valid. However, as IR procedures offer a therapeutic option, the risk of radiation exposure is usually outweighed by the benefit to the patient. IR procedures sometimes require clinically significant amounts of radiation, but the risk of radiation is low compared to other procedural risks such as haemorrhage or tissue ischaemia, while the benefits of imaging guidance are great. In fact, image-guided procedures often cause less morbidity and mortality than the equivalent surgical procedures. The potential harm due to radiation is less than the potential harm due to a procedure that is cancelled, incomplete, or clinically inadequate because of concerns over radiation.
So far, most of the process of patient selection and procedure justification in many European countries has been controlled by the Interventional Radiologists. The Interventional Radiologist is by nature committed to safety. As a professional and scientific society, the Cardiovascular and Interventional Radiological Society of Europe (CIRSE), is fully aware of its role in assuring patient safety. As a matter of fact, in its “Mission and Values” statements, CIRSE includes among its main values to be “Respectful and Safety Conscious”.

As a further example, CIRSE is actively involved in an EU Research tender: the **MEDRAPET** (**MED**ical **Ra**diation **P**rotection **E**ducation and **T**raining) project (4). The primary aim of the MEDRAPET project is the identification of needs in radiation protection training. An integrated approach to education and training with high-standard training programmes harmonised at EU level is a key prerequisite to ensure excellence in radiation protection and to implement programmes for dose optimization in medicine. CIRSE is convinced that it is essential that all stakeholders in radiation protection ensure that proper education and training are in place, in particular with regard to new technologies and complex medical exposure procedures that have been developed in the past years and that are introduced into clinical practice at a rapid pace. Correct patient referral and evaluation of alternative treatment options are the first important steps of radiation dose reduction.

Another important aspect of CIRSE commitment to safety is the “CIRSE IR Patient Safety Checklist” (5). The first three items in this list are relevant to the concept of referral and procedure planning:

1. Was the case-procedure discussed with the referring physician or within a MDT?
2. Were relevant previous imaging studies reviewed?
3. Was relevant medical history reviewed?

This concept is underlined in a recent review article on “Clinical Radiation Management for Fluoroscopically Guided Interventional Procedures” by Miller et al. (6). The authors note how pre-procedural radiation dose management starts with a multidisciplinary discussion on treatment options. It is important to realize that Interventional Radiology procedures are effectively surgical procedures and extra information is required, in terms of patient safety, than for diagnostic imaging. Coagulation status, relevant medical and past medical history, performance status, informed consent and patient and/or family expectations are all important issues that require attention before any IR procedure is scheduled.

It is during this multidisciplinary discussion that the Interventional Radiologist acts as a “gatekeeper” of referral. This may happen at multiple levels. The IR may be directly

consulted by the referring physician, over the phone or during a joint review of clinical and imaging results. IRs are increasingly seeing patients in an outpatient setting to discuss the potential IR procedure, alternatives, risks and benefits. For more complex cases, the treatment options are discussed within Multidisciplinary Teams (MDTs) and the great majority of oncologic IR procedures are discussed during Tumour Board Panels. In addition, procedures are scheduled by the Interventional Radiologist and the administrative team in adherence to currently available Practice Guidelines and applying a Level 3 justification, where the application of the specific procedure to an individual patient must be justified.

Now the question that arises is: what kind of IR Referral Guidelines are available in Europe that may assist referring physicians and the Interventional Radiologist in everyday clinical practice?

Currently in Europe, IR procedures are usually included in Referral Guidelines developed for DR. This can happen in two ways. IR procedures may be listed in a dedicated section, as is the case for the guidelines of SIRM (Italian Society of Radiology) (7) and of the Société Française de Radiologie (8); in both guidelines there is an "Interventional Radiology" subsection which lists between 35 and 50 common IR procedures. For each clinical condition and relative IR procedure the level of recommendation is indicated, together with the level of radiation dose involved (in a generic 1-4 grade scale) and a comment explaining the specific features of the procedure described.

In other Diagnostic Imaging Referral Guidelines, the IR procedures are most commonly listed as part of a diagnostic problem. This is the case, for example, of "Percutaneous Transhepatic Cholangiography" which is discussed and analyzed within the diagnostic problem "Jaundice" in the section "Gastrointestinal system", as reported in the Referral Guidelines developed by the Royal College of Radiologists (9). For each IR procedure, the level of recommendation, the level of radiation dose involved (in a 1-4 grade scale) and a comment on the specific features of the procedure are included.

In each of these two approaches, multidisciplinary knowledge was gathered in the preparation of the guidelines. Experts from several clinical specialties and several different scientific societies contributed to the creation of guidelines of member states of the European Union. Despite this effort, there is not full coverage of all the clinical conditions that can be encountered in everyday practice and the whole spectrum of IR procedures that can be performed is not analyzed in detail. We believe that a complete set of referral guidelines for IR is not possible because of the individual nature of each referral and consultation.

A different way to look at referral guidelines in IR, is to find them within multidisciplinary guidelines which are in most cases prepared through an intersociety consensus. These multidisciplinary guidelines are designed to assist physicians, patients, health-care providers, and health authorities in the decision-making processes according to evidence based data.

Several examples can be found in the literature. Management of hepatocellular carcinoma is discussed in the EASL–EORTC Clinical Practice Guidelines (10). Guidelines for the early management of the adults with ischemic stroke are given in an intersociety document prepared by North American cardiology and neurology associations (11), while guidelines for

the management of peripheral arterial disease are described in the TASC-II document whose preparation involved several scientific societies from different disciplines in Europe and North America (12).

Multidisciplinary guidelines prepared through intersociety consensus, most of the times do not mention radiation exposure issues. It would be advisable that these issues be considered in future multidisciplinary guidelines and that agencies and societies involved in radiation protection be consulted before publication.

Conclusion

Practice guidelines for IR are not fully implemented in current DR guidelines. No member state of the European Union has independent guidelines created for IR by the national IR society. In most cases, in clinical practice, the Interventional Radiologists has the role of regulating the referrals (clinical gatekeeper). At the present state, it would be really difficult to try and create guidelines for all IR procedures as risks do not just relate to radiation dose and each referral is an individual contract between the patient and the Interventional Radiologist. In any case, multidisciplinary treatment guidelines should be preferred.

References

1. International Commission on Radiological Protection. Avoidance of radiation injuries from medical interventional procedures. ICRP publication 85. Ann ICRP 2000; 30 (2): 7 – 67 .
2. National Cancer Institute. Interventional fluoroscopy: reducing radiation risks for patients and staff. NIH Publication No. 05-5286. Bethesda, MD: National Cancer Institute, 2005. http://www.cancer.gov/cancertopics/causes/radiation/interventionalfluoroscopy
3. United Nations Scientific Committee on the Effects of Atomic Radiation. Sources and effects of ionizing radiation. New York, NY: United Nations, 2000 .
4. MEDRAPET Project - http://www.medrapet.eu/
5. CIRSE IR Patient Safety Checklist - http://www.cirse.org/?pid=690
6. Clinical radiation management for fluoroscopically guided interventional procedures. DL Miller et al. Radiology 2010; 257: 321-332.
7. Referral Guidelines of SIRM - http://www.sirm.org/it/linee-guida.html
8. SFR http://www.sfrnet.org/sfr/professionnels/5-referentiels-bonnes-pratiques/guides/guide-bon-usage-examens-imagerie-medicale/index.phtml
9. i-Refer – Making the best use of clinical radiology – 7th edition 2012- The Royal College of Radiologists
10. EASL–EORTC Clinical Practice Guidelines: Management of hepatocellular carcinoma. J Hepatol 2012, 56: 908–943.
11. Guidelines for the early management of adults with ischemic stroke: a guideline from the American Heart Association/American Stroke Association Stroke Council, Clinical Cardiology Council, Cardiovascular Radiology and Intervention Council, and the Atherosclerotic Peripheral Vascular Disease and Quality of Care Outcomes in Research Interdisciplinary Working Groups. Stroke 2007; 38: 1655-711.
12. TASC-II document. Intersociety consensus for the management of PAD. http://www.tasc-2-pad.org

----

Talk 13: Referral guidelines and Nuclear Medicine

Fred Verzijlbergen, European Association of Nuclear Medicine (EANM)

Prof. Fred Verzijbergen
President of the European Association of Nuclear Medicine
j.verzijlbergen@erasmusmc.nl

The European Association of Nuclear Medicine (EANM) is comprised of 3600 individual members, 39 national societies, and has 16 affiliated members (countries outside Council of Europe). The governance structure for EANM is shown in the figure below:

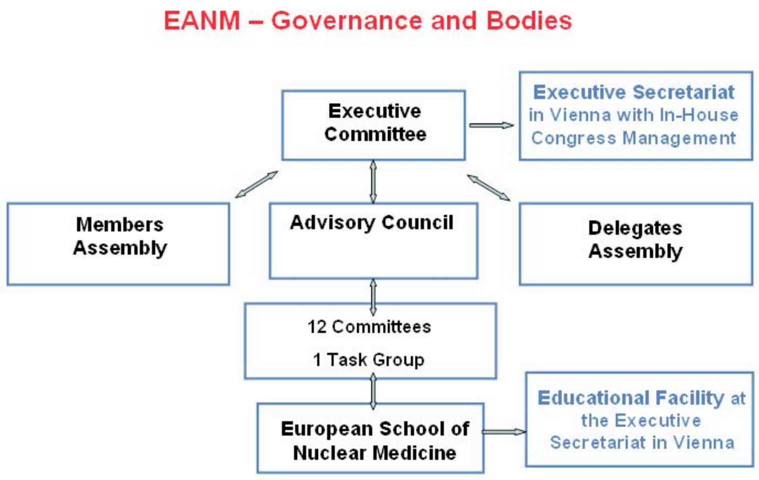

Of the twelve EANM committees, the **Dosimetry Committee** has published several documents, including:

1. A Series on Standard Operational Procedures for Pre-Therapeutic Dosimetry II. Radioiodine Test Prior to Radioiodine Therapy of Benign Thyroid Disease (revision 2012 in progress)
2. A guidance document on good practice of clinical dosimetry reporting (publication: 2010)
3. Guidelines for bone marrow and whole-body dosimetry (publication: 2010)
4. A series on standard operational procedures for pre-therapeutic dosimetry: blood and bone marrow dosimetry in differentiated thyroid cancer therapy (publication: 2008)

The **Paediatrics Committee** has published the following guidelines:

1. Guidelines for standard and diuretic renogram in children **(**publication: 2011)
2. Guideline for radioiodinated MIBG scintigraphy in children (publication: 2011)
3. Guidelines for paediatric bone scanning with 99mTc-labelled radiopharmaceuticals and 18F-fluoride (publication: 2010)
4. Guidelines on 99mTc-DMSA Scintigraphy in children (publication: 1st version 2001, revised version 2009)
5. Guidelines for lung scintigraphy in children (publication: 2007)
6. Guidelines for 18F-FDG PET and PET-CT imaging in paediatric oncology (date of publication not-available)

The **Cardiovascular Committee** has published the following:

1. Hybrid cardiac imaging: SPECT/CT and PET/CT. A joint position statement by the European Association of Nuclear Medicine (EANM), the European Society of Cardiac Radiology (ESCR) and the European Council of Nuclear Cardiology (ECNC) - (publication: 2010)
2. EANM/ESC guidelines for radionuclide imaging of cardiac function (publication: 2008)
3. EANM/ESC procedural guidelines for myocardial perfusion imaging in nuclear cardiology (publication: 2005, currently under revision)

The **Oncology Committee** has published the following:

1. PET in radiotherapy planning: Particularly exquisite test or pending and experimental tool? (publication by EANM and ESTRO 2010)
2. EANM-EORTC general recommendations for sentinel node diagnostics in melanoma (publication: 2009)
3. Joint practice guidelines for radionuclide lymphoscintigraphy for sentinel node localization in oral/oropharyngeal squamous cell carcinoma (publication: 2009)
4. FDG PET and PET/CT: EANM procedure guidelines for tumour PET imaging: version 1.0 (publication: 2009)
5. Sentinel node in breast cancer procedural guidelines (publication: 2007)

Do the guidelines work?

- Most European NM physicians check the request the day before the actual study Pharmacists and Physicists are members of the team
- Working according to guidelines is a constant issue during audits
- Further improvement is required on the clinicians' side

Talk 14: Radiation dose issues and risk

Reinhard Loose, German Commission on Radiological Protection (SSK)

Reinhard Loose MD PhD
German Commission on Radiological Protection (SSK)
German Roentgen Society (DRG)
loose@klinikum-nuernberg.de

Justification is the first step in diagnostic radiology before applying ionizing radiation to an individual patient. This first step should always be a balance between the expected benefit and the risk of radiation. Depending on the type of imaging procedure the dose is variable in a range of more than 1:1000. Typical dose values for X-ray examinations are listed in Table 1.

EU (RP 118)			
Radiographic examinations:	mSv	# chest exams	# days natural background
Limbs and joints (except hip)	<0.01	<0.5	<1.5 days
Chest (single PA film)	0.02	1	3 days
Skull	0.06	3	9 days
Thoracic spine	0.7	35	4 months
Lumbar spine	1.0	50	5 months
Hip	0.4	20	2 months
Pelvis	0.7	35	4 months
Abdomen	0.7	35	4 months
IVU	2.4	120	14 months
Barium swallow	1.5	75	8 months
Barium meal	2.6	130	15 months
Barium follow through	3	150	16 months
Barium enema	7.2	360	3.2 years
CT head	2.0	100	10 months
CT chest	8	400	3.6 years
CT abdomen or pelvis	10	500	4.5 years

Table 1: typical effective doses for radiography, fluoroscopy and CT examinations, comparison with number of single PA chest examinations and comparison with natural background exposure (EU – RP 118) [1].

The next question is how reliable our data are and where they come from?
Table 2 shows dose values for radiographic chest examinations in different countries. It cannot be explained with the physical patient properties why the ratio of pa/lat view in Switzerland is 1:2 and in Belgium 1:8 (blue) or why the DAP for a pa view in Germany is about 20% of Norway (green). Hence, in future a more standardized approach for data acquisition is mandatory.

SOURCES AND EFFECTS OF IONIZING RADIATION

United Nations Scientific Committee on the Effects of Atomic Radiation

UNSCEAR 2008
Report to the General Assembly
with Scientific Annexes

VOLUME I

How reliable are our data ?

- **Questionnaires ?**
- **Health insurance companies ?**
- **National reporting systems ?**

Country	Chest		
	Chest PA	Chest LAT	
Australia	0.16	0.73	
Belgium	0.15	1.23	ratio pa/lat 1:8.2
Czech Republic	0.40	1.20	
Germany	**0.13**	0.46	DAP pa 20% of Norway
Greece	0.50		
Hungary	0.52	0.91	
Iceland	**0.57**		
Japan	0.33	0.44	
Lithuania	0.44	1.60	
Malta	0.20	0.45	
Netherlands	0.04		
Norway	**0.64**	**0.82**	DAP pa 0.64 µGy cm^2
Romania	1.30	3.50	
Slovenia	0.29	0.96	
Spain	0.17	0.49	
Sweden	**0.40**	**0.40**	
Switzerland	0.10	0.20	ratio pa/lat 1:2.0
United Kingdom	0.16		

Table 2: Dose comparison for chest radiography in different countries. Regular type values are entrance air kerma in mGy, bold type values are for DAP in Gy cm^2 (UNSCEAR 2008).

Band Classification of the typical effective doses of ionising radiation from common imaging procedures

Band	Typical effective dose (mSv)	Examples
0	0	US, MRI
I	<1	CXR, XR limb, XR pelvis
II*	1-5	IVU, XR lumbar spine, NM (e.g. skeletal scintigram), CT head & neck
III	5-10	CT chest and abdomen, NM (e.g. cardiac)
IV	>10	Some NM studies (e.g. some PET)

GP → Radiologist: Level of justification

* *The average annual background dose in most parts of Europe falls in band II.*

Table 3: Dose bands for different groups of radiological examinations (UNSCEAR 2008)

Due to the wide dose range of all available radiological examinations it is helpful for justification to define dose bands from 0 (US and MRI), I (simple radiographic images) up to III and IV with CT of the trunk, PET-CT and some nuclear medicine studies. Dose band I is the range of natural radiation dose or below and needs a simple individual justification, which is in most cases included in the medical question of the referring physician. Examinations

within the dose bands III and IV require a careful individual justification by a radiologist. In some cases it may be necessary to contact the referrer and change the requested procedure.

The associated risks of most medical X-ray examinations are comparable with other risks of daily life like smoking, car driving, sports and many others.

A significant difference between risks of daily life and medical imaging is the distribution of dose as a function of age and severity of disease. Figure 1 shows the dose distribution of 403 randomized patients in a large teaching hospital (Nuremberg). About 50% of patients get no X-ray examinations whereas in the exposed group 44 patients receive 90% and 10 patients 50% of the collective dose [3]. This demonstrates clearly that a minority of patients with high disease related risks receive the predominant portion of dose.

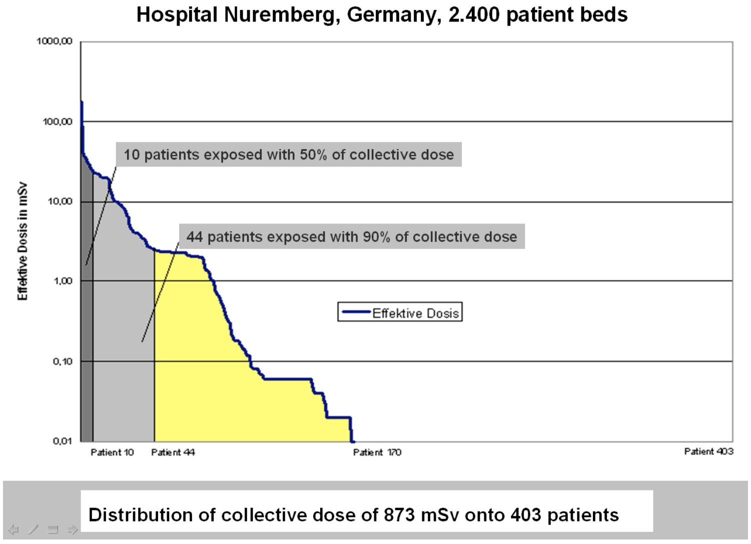

Figure 1: Distribution of collective X-ray dose on 403 randomized patients

References

1. RADIATION PROTECTION 118, Referral guidelines for imaging, Office for Official Publications of the European Communities, 2001. ISBN 92-828-9454-1
2. SOURCES AND EFFECTS OF IONIZING RADIANTION. United Nations Scientific Committee on the Effects of Atomic Radiation. UNSCEAR 2008, Volume 1, ISBN 978-92-1-142274-0
3. R. W. Loose, U. Popp, M. Wucherer, R. Adamus. Medical Radiation Exposure and Justification at a Large Teaching Hospital: Comparison of Radiation-Related and Disease-Related Risks. Fortschr Röntgenstr (RoeFo) 2010; 182: 66 –70

Talk 15: Imaging referral guidelines and implementation issues

Steve Ebdon-Jackson, Radiation Protection Division, UK HPA

Steve Ebdon-Jackson
Head, Medical Exposure Department, Radiation Protection Division, Health Protection Agency, UK
Steve.ebdon-jackson@hpa.org.uk

Euratom Directives establishing frameworks for radiation protection have been, for many years, the basis for regulations in European Member States. These have laid down the basic measures for the health protection of individuals against the dangers associated with ionising radiation. It is perhaps surprising therefore that Medical Exposure Directive 84/466/Euratom[1] made no reference to referrers or to referral guidelines. As a consequence, national regulations such as the UK's POPUMET regulations[2] made no reference to them either.

This did not prevent professional bodies and organisations taking a lead in these matters. In 1989, the Royal College of Radiologists (RCR) produced the first of a series of guidelines "Making the Best Use of a Department of Clinical Radiology"[3], the latest of which was issued in 2012[4].In 1990, the RCR, in conjunction with the UK's National Radiological Protection Board (NRPB) issued its document "Patient dose reduction in diagnostic radiology"[5] which highlighted that up to 20% of radiology procedures in the UK might be clinically unhelpful.

The Department of Health (DH) in England was broadly supportive of the RCR's approach, but recognised the costs and logistics involved in production and dissemination. Nevertheless, such was the importance of the initiative, DH began to consider ways in which the need for referral guidelines could be incorporated into regulations.

The basis for doing so was provided by Directive 97/43/Euratom[6] which required Member States to ensure that recommendations concerning referral criteria were available to those who referred patients for medical exposures and in Great Britain this was implemented in the Ionising Radiation (Medical Exposure) Regulations 2000[7].

This was accepted by professions and hospital management without opposition; the RCR guidelines were already in their 4th edition. These guidelines were in use in every UK hospital and provided a standard of care. This has continued and in the edition of 2012, the change of name to "iRefer" demonstrates a maturity of development and confidence of the importance of such guidelines.

Inevitably, questions have been raised as to use rather than availability and value of guidelines within the referral and justification processes. Inclusion within a regulatory package should ensure implementation, but evidence of this is best provided through local and national audit. One such study in Sweden[8] has demonstrated that the impact of guidelines might be less than expected, with the conclusion that 20% of CT examinations are not justified. Further examination of the situation however shows that the guidelines in place at that time were those produced by the European Commission in 2001[9]. Lack of up to date and locally derived guidelines may have influenced local practice.

Experience of use of guidelines does show that their use takes time and professional resolve. It might be easier to agree to referrals without question and to ignore the guidelines in place.

This is a matter of professional integrity. Experience of production of guidelines has shown that the process in expensive and time consuming, if it is done well and if guidelines are to have an acceptable level of evidence base. Nevertheless, despite these issues, there remains a role for referral guidelines, as long as they are up to date and auditing shows their use. The costs may not be trivial, but the potential impact is significant and it is important that funding is secured to ensure the continuing development and evolution of referral guidelines.

References

1. Council Directive 84/466/Euratom Off J Eur Commun, No 265 5.10.84
2. The Ionising Radiation (Protection of Persons Undergoing Medical Exposure) Regulations 1988 (SI 1988 No. 778) London HMSO
3. Making the Best Use of a Department of Clinical Radiology Royal College of Radiologists 1989
4. iRefer Making the best use of clinical radiology Royal College of Radiologists 2012
5. Patient Dose Reduction in Diagnostic Radiology National Radiological Protection Board Vol 1 No 3 1990
6. Council Directive 97/43/Euratom Off J Eur Commun, No L180 9.7.97
7. The Ionising Radiation (Medical Exposure) Regulations 2000 (SI 2000 No. 1059) London HMSO
8. National Survey on Justification of CT examinations in Sweden SSI 2009:03
9. Referral Guidelines for Imaging European Commission 2001 RP 118

Talk 16: Imaging referral guidelines and radiography

Graciano Paulo, European Federation of Radiographer Societies (EFRS)

Prof. Graciano Paulo, President of the European Federation of Radiographer Societies
graciano@estescoimbra.pt

Since Roentgens' discovery professionals have obtained medical images for clinical diagnostic purposes. This can be accounted to be the origin of Radiographers' profession: a health professional that acts as the interface between patient and technology. In November 2011, the EFRS General Assembly approved a definition for the term "Radiographer" to be used in all international documents, to avoid further confusion and misinterpretation arising from the fact that there are several names referring to the same profession across Europe.

Therefore and according to the approved definition, Radiographers are recognised as being medical imaging and radiotherapy experts who:

- Are professionally accountable to the patients' physical and psychological well being, prior to, during and following examinations or therapy;
- Take an active role in the justification and optimization of medical imaging and radio therapeutic procedures;
- Are key-persons in the radiation safety of patients and third persons in accordance with the "As Low As Reasonably Achievable" (ALARA) principal and relevant legislation.

It is expected that the radiographer will have professional autonomy and accountability, develop good professional relationships, develop personal and professional skills and

demonstrate an ethical and knowledgeable understanding of the profession. It is considered vital that professional advancement arises out of evidence-based practice and is informed through focused research.

To guide these expectations EFRS has created a European Code of Ethics for Radiographers, focused on the radiographers' professional life, relation with the patients and personal and professional standards.

The outstanding evolution of imaging technology gave health systems, the capability to produce more and faster imaging procedures. Behind this "more and faster" concept, a progressive increase of patient dose and exam frequency (especially those delivering higher dose, such as CT and interventional procedures) is unfortunately verified.

Published data indicates reasons for this phenomenon, such as: lack of dose exposure awareness by referring practitioners; defensive medicine practice; out-dated information regarding new modalities or technological solutions; lack of a centralized patient data center policy implementation by the National Health Service (NHS) of each country, to avoid exam repetition; major cultural and organizational differences between each country NHS; patient pressure to perform more procedures; etc.

Health systems are complex socio-technical environments sustained on multi-professional teams, affecting and be affected by several variables that dramatically influence daily practice, such as:

- asymmetric knowledge (even within the same profession);
- some working in primary care units and others at highly technical equipped hospitals;
- some with easy access to all kind of technology and others limited to basic diagnostic tools and with difficulties in referring;
- some working in a "pure" NHS model (with almost 100% of health care delivery in public institutions) and others working simultaneously in public and private practice;

It's in this complex environment that solutions must be found to develop, create, disseminate and audit the implementation of EU imaging referral guidelines (IRGL). EFRS supports that IRGL are an important tool to drive clinical practice towards effective, efficient and safe patient care delivery, according to evidence-based standards.

It's important to be aware that IRGL are not only about Patient Radiation Protection, but also about prescribing (if necessary) the most adequate medical imaging procedure, according to the patient's clinical signs, symptoms and needs. These guidelines, once implemented, will certainly contribute to decrease medical imaging procedures and consequently decrease exposure, health expenditure and patient waiting list.

IRGL will help to build a bridge between science and daily practice. However, professional societies should be aware that one of the most important tools for the success of its implementation is the development of a perfect communication tool to deliver information to its members.

A more effective regulation of health systems and professional practice is also needed for the success of the implementation of IRGL, as well as a combined strategy to implement an

effective clinical audit system, as a tool for promoting patient safety culture and improve the quality of care.

Therefore, there are several challenges for European IRGL. They have to adapt to professional daily practice and to each national health system model and be creative in finding a mechanism that avoids conflicts between health professionals, in regard to justification processes in medical imaging, due to:

- different professional opinions;
- economical impact of those opinions;
- patient rights

Due to the role of Radiographers' in a modern health care system, daily base practice demonstrates that they play a key role in medical imaging procedures, acting as an interface between patient and technology.

Radiographers are the pivot between referrers, patients and radiologists, and therefore a key player in the implementation of IRGL.

Being the final point of contact for the patient, Radiographers have the responsibility to guarantee the correct procedure to the right patient while ensuring maximum optimization of the procedure.

EFRS, as representative of European Radiographers, is ready for the challenge and available to be part of the solution to develop a better health system for EU citizens.

References

1. http://www.efrs.eu/the-profession/ (last accessed 07/10/2012)
2. European Code of Ethics for Radiographers, EFRS 2010
3. Overview of the Tuning Template for Radiography in Europe, http://www.unideusto.org/tuningeu/images/stories/template/Radiography_overview.pdf (last accesses 18/10/2012)
4. UNSCEAR 2008 report. Sources and effects of ionizing radiation. Published in 2010. Available on http://www.unscear.org/unscear/en/publications.html (last accessed 19/10/2012).

Talk 17: Imaging referral guidelines for general practitioners
Wolfgang Spiegel, Austrian Society of General Practice and Family Medicine

Wolfgang Spiegel, MD
Department of General Practice, Center for Public Health, Medical University of Vienna, Austria
Wolfgang.spiegel@meduniwien.ac.at

What GPs imagine radiological reports to be and what they wish from radiologists

Keep it clear and short:

- The referral question should be clearly addressed.
- Always give a brief summary of the radiological findings at the end of the report.

- State opinion about the dignity or clinical relevance of the main radiological findings.

What makes life easier for GPs:
Radiologists, please kindly:

- Give a first interpretation of the radiological test to the patient (if the referring GP likes you to do that) but do not address treatment options or patient management which is the duty of the GP.
- Get in contact with GP if immediate action is needed (e.g. a fracture) to coordinate patient management.
- Get in contact with GP if bad news needs to be broken to patient.
- Do not use abbreviations (acronyms).
- Do not say "due to gas in intestine assessment of the pancreas not possible". Assure quality.
- If quality of examination is not given (e.g. gas in intestine) reschedule and improve patient preparation.

What GPs would like radiologists rather not to do:

- Do not make any routine statement about when the test should be repeated!
- Do not recommend additional radiological imaging methods (e.g. MRI) in test report unless really unavoidable.
- If additional radiological imaging is indicated consider personal communication (e.g. telephone) with physician who referred patient.
- Do not tell the patient to which specialist he/she needs to go. Refer patient back to GP.

What radiologists are entitled to expect from GPs/FPs:

GPs/FPs:

- have to assess the need (indication) for radiological testing carefully.
- have to clearly state the clinical question.
- need to evaluate risk (e.g. radiation) and possible benefit (if available according to guidelines).

Radiologists can expect GPs/FPs:

- to inform them about the patient's clinical conditions and physical findings (referral letter or form).
- that they inform their patient about the sense of proposed radiological testing.
- that they interpret radiological findings to the patient with care and assure optimal patient management in the given setting.

What makes life easier for radiologists – a GP's view:

GPs/FPs:

- should brief radiologists about their practise style and possible spectrum of care.
- should inform radiologist beforehand about desired patient management when immediate action might be required after imaging is done.

What GPs should rather not do:

GPs/FPs:

- should not give in to patients' demands on radiological testing without clear indication (reason for radiological testing).

Summary and conclusion:
Good communication between GPs and radiologists is essential for efficient and patient-oriented use of imaging methods. Both GPs and radiologists have professional principles and needs and expect each other to take them into account in professional interactions.

Guidelines and recommendations

Council Directive 97/EURATOM *ARTICLE 3, JUSTIFICATION*
1. Medical exposure referred to in Article 1 (2) shall show a sufficient net benefit, weighing the total potential diagnostic or therapeutic benefits it produces, including the direct health benefits to an individual and the benefits to society, against the individual detriment that the exposure might cause, taking into account the efficacy, benefits and risks of available alternative techniques having the same objective but involving no or less exposure to ionizing radiation.

Council Directive 97/EURATOM *ARTICLE 5, RESPONSIBILITIES*

1. The prescriber as well as the practitioner shall be involved as specified by Member States in the justification process at the appropriate level.
2. Member States shall ensure that any medical exposure referred to in Article 1 (2) is effected under the clinical responsibility of a practitioner.

Conclusions
Introduction of radiological guidelines together with feedback on referral rates was effective in reducing the number of requests for spinal examinations over one year. Wider use of GP-orientated guidelines with regular updating and feedback might save costs and reduce unnecessary irradiation of patients.

Summary

- Communication between GPs and radiologists is essential for patient-oriented use of imaging methods.
- Both GPs and radiologists have professional needs and expect each other to take them into account in professional interactions.
- It improves coordination of care if GPs brief referral radiologist of possible patient management strategies in case of critical findings.

Talk 18: The patient's perspective

Alison Meyric-Hughes, Patient Liaison Group, The Royal College of Radiologists (RCR)

A.H.Meyric-Hughes@city.ac.uk

Many people are anxious when going to an imaging unit, both about the procedure itself and what it might reveal. Information about what happens before, during and after a procedure, including when and how the results will be given, helps allay patients' fears.

Referrers must check how patients will receive the necessary information. It is the imaging service that is responsible for creating and sending up-to-date, patient friendly guidelines and information. This needs to be written in clear, plain language without the use of acronyms. It should be available in a variety of formats, including, for example, Braille, and in the various languages appropriate to the location of the service.

Patients need to be told by the referrer why a service has been chosen, which is sometimes not the local hospital, and why the particular procedure has been selected.

Citing the referral guidelines can be useful in gaining patients' confidence and in reducing the feeling of lack of control. This, and the points made above, should help patients understand their conditions and give them a measure of sense of control. Referrers should remember the mantra "no decision about me without me".
