# Supplementary material for: Imaging referral guidelines in Europe: now and in the future—EC Referral Guidelines Workshop Proceedings
Source: Insights Imaging. 2013 Dec 13;5(1):9–13. doi: 10.1007/s13244-013-0299-8 (PMC3948903; doi:10.1007/s13244-013-0299-8)
Supplement: Supplementary file 3 — Workshop proceedings, Session 3 Survey Feedback (PDF 110 kb) [file 13244_2013_299_MOESM3_ESM.pdf]

Project ENER/D4/315-2011
Implementation of Council Directive 97/43/Euratom requirements concerning referral criteria for medical imaging in the European Union

IMAGING REFERRAL GUIDELINES IN EUROPE: NOW AND THE FUTURE.
EC REFERRAL GUIDELINES WORKSHOP
Billrothhaus, Vienna.
September 20-21 2012

Workshop Proceedings

Session 3: Survey Feedback

Chair: Jean-François Chateil, European Society of Paediatric Radiology (ESPR)
Rapporteur: Steve Ebdon-Jackson, Radiation Protection Division, UK HPA

Talk 19: Guidelines survey development

Nick Ashford, The Royal College of Radiologists (RCR)

Dr. Nick Ashford, Treasurer of the Royal College of Radiologists, UK
Nick.Ashford@wsht.nhs.uk

A questionnaire was developed by the RCR during December and January 2011-2012 following submission of the proposal for the project "Implementation of Council Directive 97/43/Euratom requirements concerning referral criteria for medical imaging in the European Union" as submitted as a consortium in June 2011.

A web-based survey was used to assess the availability of imaging referral guidelines, developmental methodology, preferences for future initiatives for European community action to facilitate justification and appropriate use of radiological diagnostic procedures
An Initial draft was devised by the RCR, by a team which included: Denis Remedios, Pete Cavanagh, Hazel Beckett, Bethan Seymour and Nick Ashford, and this was presented at the Referral Guidelines Tender Project Kick-Off Meeting on 19th January 2012 in Luxembourg;

- Implementation of Council Directive 97/43/Euratom requirements concerning referral criteria for medical
- Imaging in the European Union
- EC Tender Contract no. ENER / 11 /NUCL/S12.6O691 5

The draft was subsequently developed further in WP1 with input from all the members of the project consortium*:*

- European Society of Radiology (ESR) , Project Coordinator
- The Royal College of Radiologists (RCR)
- Société Française de Radiologie (SFR)
- Cardiovascular and Interventional Radiological Society of Europe (CIRSE)
- European Society for Paediatric Radiology (ESPR)
- European Commission, DG Energy

The individual members were:

- Georgi Simeonov, European Commission
- Denis Remedios, ESR
- Philippe Grenier, SFR
- Mario Bezzi, CIRSE
- Karen Rosendahl, ESPR
- Maria Del Rosario Perez, WHO, external expert
- Madan Rehani, IAEA, external expert
- Monika Hierath, Project Manager, ESR
- Nick Ashford and Pete Cavanagh RCR

The survey design was presented at the EC referral Guidelines Workshop September 21st in Vienna. Important points included:

- Guidelines Design of survey
- Methodology of guidelines
- Guideline availability and distribution
- Funding of guidelines
- Audit
- Barriers
- Solutions
- Bonus questions
- Future wishes

The design of the study was illustrated by discussing a number of the key survey questions and points.

The web based survey, compiled in SurveyMonkey™**,** was distributed to national radiological societies, national nuclear medicine societies and national radiological protection competent authorities. The survey was designed to be easily understood with carefully chosen simple terminology, easy to analyse statistically with few free text answers, and easy to complete with seamless design leading responders only through the relevant questions. A Likert scale was used to quantify the answers and to explore the way forward.

The questionnaire was meticulously tested and further discussed in Vienna on March 5th before being distributed by the ESR and then kindly analysed by the SFR.

A selection of the survey questions including the core questions were presented at the Final Workshop to demonstrate the content, style, aims and methodology of the survey.

In conclusion, the intention of this presentation and summary was to clearly demonstrate how a complex and comprehensive survey, designed by the RCR with support from the Project Consortium, could also be easy to complete.

Talk 20: Guidelines survey and analysis of questionnaire
Valérie Vilgrain, the Société Française de Radiologie (SFR)

Dr. Valerie Vilgrain is Chair of the Department of Radiology at the University Beaujon Hospital, Clichy, Paris 7 University, France
valerie.vilgrain@bjn.aphp.fr

A web-based survey was used to assess the availability of imaging referral guidelines, development methodology, and the preferences for future initiatives for European community action to facilitate justification and appropriate use of radiological diagnostic procedures. A questionnaire devised by the RCR together with other members of the project consortium was distributed to representatives of national radiological and nuclear medicine societies as well as national radiological protection competent authorities in 30 European countries including 27 European Union member states, Croatia (the candidate country) and countries using European legislation viz. Norway and Switzerland. Responses were collated by the ESR and analysed by the SFR together with the Steering Committee.

Analysis of responses to the questions 1 to 12

Thirty countries responded and 80 responses were received. Of these:

- 32 were from national radiological societies
- 20 were from national nuclear medicine societies
- 28 were from national competent authorities

From the national radiological societies, no response was received from Cyprus, two responses were received from the Netherlands and three from Romania.

From the national nuclear medicine societies, no response was received from Bulgaria, Estonia, Finland, France, Ireland, Lithuania, Luxembourg, Norway, Poland, Portugal, Slovakia or Slovenia. Three responses were received from the Netherlands.

From the national competent authorities, responses were received from all countries, except Italia, Hungary, Latvia and two responses were received from Spain.

In the analysis, all the responses received were taken into account as it was impossible to select or to merge the different responses coming from the same countries.

61% of responders said “yes: There is a legal requirement for imaging in referral guidelines including radiation dose”. Interestingly, 5 responders said: “I don’t know whether such a legal requirement exists or not”.

Surprisingly, there were some disagreements between national societies and competent authorities’ responses to the question about the transfer responsibility for making guidelines availability. 17 national societies answered “I don’t know” versus one competent authority.

In the great majority of responses, the responsibility for making guidelines has been transferred to department/ministry of health, or professional organizations.

The great majority of states have recommended European or national guidelines.

The great majority (76%) of responders answered “No” to the question concerning the insurance requirements stating that a guideline must exist in order for there to be a payment for an imaging investigation (10% answered “I don’t know”).

Regarding which professionals make requests, most of them are made by medical doctors. Among the medical doctors, General Practitioners differed from emergency dept. Clinicians and specialist/hospital doctors as the former essentially make requests for plain radiography, contrast radiography, and ultrasound. According to national societies’ responses, GPs more rarely make requests for CT, MRI, IR and nuclear medicine examinations.

Analysis of responses to the questions 13 to 41

20 radiology societies and 12 nuclear medicine societies, and surprisingly only 8 competent authorities, responded that there are nationally recognized imaging referral guidelines including radiation dose available.

Only the responses from the national societies were taken into consideration as the number of competent authorities which responded was relatively small. The responses from the competent authorities to this question were often incomplete and sometimes discordant with those of national societies.

Only 12 of the 20 radiological societies and 9 of the 12 nuclear medicine societies having nationally recognized imaging referral guidelines including radiation dose answered the subsequent questions concerning specifically the content of these guidelines. These guidelines were issued from a single source for 4 national societies and from multiple sources for 11.

The guidelines were nationally developed in half of the countries and modified or adopted with modifications from another source in the others. There was a good correspondence between radiology and nuclear medicine societies taking into account two countries (France and UK) which have delivered radiological and NM guidelines in the same document.

The year of the first edition varied from 1989 to 2005 for radiological societies and from 1998 to 2011 for nuclear medicine societies. The approximate duration of the review cycle has varied between countries from 3-4 years to > 6 years.

In the majority of countries (67% for radiology and 90% for nuclear medicine), the source of funding was departments or ministries of health or other governmental departments.

The radiating imaging modalities (RX and nuclear medicine) were included in the great majority of the guidelines (83-92%) whereas the non radiating imaging modalities (US, MRI) were only present in 75% of the guidelines.

The majority of guidelines have produced separate guidance for children (67-80%) and pregnant women (83-78%).

Almost all radiological societies' guidelines have covered all groups of medical conditions. It has been often less covered in nuclear medicine guidelines.

Only 7 of 12 guidelines of radiological societies and 2 of 11 nuclear medicine societies have been based on clinical presentations and their imaging investigations.

Radiation dose, strength of evidence and grading of recommendations were considered in all or almost all guidelines. Cost effectiveness and availability of equipment or expertise were much less often considered.

Very few guidelines have included recognized evidence levels (6 radiology, 3 nuclear medicine) and grading recommendation using a recognized system (4 radiology, 1 nuclear medicine).

Delphi process was used in 3 radiological societies' guidelines (Finland, France, UK). Expert meeting for consensus was used by 4 radiological societies and 5 nuclear medicine societies.

Radiation dose was obtained from recognized source in 8 radiological and 9 nuclear medicine societies.

The radiological societies' guidelines included between 200 to 500 clinical conditions or diagnostic problems, and the nuclear medicine societies guidelines between 16 and 300.

Two radiological societies graded their recommendations (France and UK). The vast majority of recommendations were either B or C.

Almost all guidelines are available on downloaded digital version. The great majority of guidelines are available in a web version. Very few have a PDA/tablets or smart phones application.

The majority of guidelines routinely circulate to providers of the service, general practitioners, emergency department clinicians and specialists / hospital doctors. Only few of these guidelines routinely circulate to non healthcare professional, medical students, funders and public.

Reinforcement of guidelines is made by periodic reminders in half of guidelines and by educational message in most of the radiological societies.

Only two national societies have incorporated their guidelines into clinical decision support systems (CDSS) (Finland, Italy).

Guidelines have been mainly used for education and academic/research purposes.

Analysis of responses to the questions 42 to 47

28 responses were received from the radiology societies, 18 for NM societies and 27 from competent authorities, and all responses were taken into account. The percentage of responses rated 5-7 with a threshold of 75% were considered.

82% of radiology societies and 78% of competent authorities support European guidelines developed by combination of multiple national guidelines agreed by consensus. This is also supported to a lesser extent by nuclear medicine societies (61%). 75% of radiology societies support Pan-European guidelines developed centrally.

Most societies and competent authorities support tabular and flowchart format for the guidelines.

Most societies and competent authorities support web version (not password protected) for distribution mode. 75% of radiology societies support provision of guidelines through electronic requesting systems as a future development.

No potential barriers, or challenges to the availability of guidelines, exceed the threshold of 75% in responses rated 5-7.

Education and involvement of referring clinicians are mostly proposed by competent authorities to solve barriers limiting the availability of guidelines.

Competent authorities strongly support local internal and external clinical audits to monitor guideline use.

Key Points

1. The results of this survey might not be completely accurate as some countries responded several times and others have pooled results with radiological societies and nuclear medicine societies.
2. This survey shows that few countries have developed national guidelines including radiation dose.
3. Not all national guidelines available are based on clinical indications.
4. No significant barriers or major challenges to the development of guidelines have been raised by national societies or competent authorities.
5. The majority of responders are supporting the development of European guidelines either developed by combination of multiple national guidelines agreed by consensus or Pan-European developed centrally.
6. There is a trend to support the concept of integrating guidelines into clinical decision support systems.

Talk 21: Good practices in Europe
National radiology societies, competent authority representatives

A number of presentations of good practice were made by competent authorities and members of professional societies including Finland, Croatia, Germany, Greece and Norway. These provided examples of different approaches, support and content including the availability of national guidelines for paediatric examinations, better and improving coordination between professional bodies and government, the value of finance from government, with acknowledgement that some States had managed to produce guidelines without national funding and the importance of a multi-disciplinary approach, including the end user.
