# Supplementary material for: Imaging referral guidelines in Europe: now and in the future—EC Referral Guidelines Workshop Proceedings
Source: Insights Imaging. 2013 Dec 13;5(1):9–13. doi: 10.1007/s13244-013-0299-8 (PMC3948903; doi:10.1007/s13244-013-0299-8)
Supplement: Supplementary file 4 — Workshop proceedings, Session 4 Innovations (PDF 714 kb) [file 13244_2013_299_MOESM4_ESM.pdf]

Project ENER/D4/315-2011
Implementation of Council Directive 97/43/Euratom requirements concerning referral criteria for medical imaging in the European Union

IMAGING REFERRAL GUIDELINES IN EUROPE: NOW AND THE FUTURE. EC REFERRAL GUIDELINES WORKSHOP Billrothhaus, Vienna. September 20-21 2012

Workshop Proceedings

Session 4: Innovations

Chair: Guy Frija, European Society of Radiology (ESR)
Rapporteur: Fred Verzijlbergen, European Association of Nuclear Medicine (EANM)

Talk 22: Innovations for improving Guideline use

Myriam Hunink, European Society of Radiology (ESR)

MG Myriam Hunink, MD, PhD
Radiology, Clinical Epidemiology, Decision Sciences,
Erasmus University Rotterdam & Harvard University Boston
m.hunink@erasmusmc.nl

Background and Rationale

Given the rapid pace of technological advances, the wide variety of diagnostic and therapeutic possibilities, and the continual changing evidence, the modern physician is faced with a huge amount of information that needs to be assimilated in order to make wise diagnostic and therapeutic decisions. The information overload can form an insurmountable obstacle to the individual physician. Referral guidelines can guide the physician in making smart choices and can streamline clinical practice. This is particularly true for decisions regarding imaging procedures.

Nevertheless, physicians are reluctant to use guidelines. Changing physician behaviour is notoriously difficult. Is it because they cherish their autonomy? Are they set in their ways? Is it financial incentives? Are the guidelines unavailable at the point-of-care? Or do they feel that a one-size-fits-all guideline simply does not fit their unique patient?

State-of-the-art

A systematic review of interventions to improve outpatient referrals from primary care to secondary care [1] demonstrated the paucity of rigorous evaluations in the field. Seventeen studies involving 23 separate comparisons were included in the review. Nine studies (14 comparisons) evaluated professional educational interventions.

Interventions that were ineffective included:

- passive dissemination of local referral guidelines
- feedback of referral rates
- discussion with independent medical adviser

Generally effective interventions included:

- dissemination of guidelines with structured referral sheets
- involvement of consultants (secondary care specialists) in educational activities

Promising interventions were:

- organizational interventions (examples: patient management by family physicians instead of internists, physiotherapist incorporated in general practice, a new slot system for referrals requiring a 'in-house' second opinion prior to referral)
- financial incentives (example: mixed capitation and fee-for-service system with an element of risk sharing for secondary care services)

Shortcomings

Guidelines are commonly:

- inconsistent and complex
- not integrated with other diagnostic procedures
- time-consuming to produce
- costly
- difficult to implement
- not available at the point of care
- not personalized / not patient tailored
- not setting specific
- quickly outdated

Innovations

Clinical decision support Solutions

Osheroff (2006) [2], defines it as “Clinical decision support (CDS) systems provide clinicians, staff, patients, and other individuals with knowledge and person specific information, intelligently filtered and presented at appropriate times, to enhance health and health care.”

Knowledge-based CDS systems consist of:

- a knowledge base
- an inference engine
- a communication tool

Non-Knowledge-based CDS systems include:

- artificial intelligence

- machine learning
- artificial networks

Radiology order entry with decision support: initial clinical experience [3,4,5]

- "Computerized order entry with decision support can be widely accepted by clinicians and can have an impact on ordering practices." (MGH Experience)
- Radiology order entry handles 90% of all pre-scheduled outpatient exams
- 95% of primary care physicians either use radiology order entry directly or have their clinical staff do it for them
- 80% of general Internal Medicine orders come directly from physicians
- ER CT pulmonary angiography utilization decreased by 20% whereas the yield increased from 6% to 10%.

Prediction rules

- Clinical prediction rules (prior to performing an imaging test) estimate the probability of disease conditional on clinical parameters and can thus be useful in justifying the need for an imaging procedure
- Diagnostic prediction rules (after the imaging test has been performed) estimate the probability of disease conditional on clinical parameters combined with the imaging findings and can thus be useful in guiding treatment choices based on the integrated information from all diagnostic procedures

Vision

A clinical decision support system to optimize the use of imaging would have to fulfil the following criteria:

- online clinical decision support system
- available at the point-of-care
- integrated in radiology order entry system and hospital information system
- integrated with other diagnostic procedures and diagnostic information
- linked to electronic patient record
- provides evidence-based information
- includes individual patient-tailored decision support
- both pre-test and post-test decision support
- Web 2.0 / wiki-type environment to get input from multiple stakeholders
- setting specific
- continually and dynamically updated
- adaptive Bayesian design
- self-learning

References

1. Akbari A, Mayhew A, Al-Alawi MA, Grimshaw J, Winkens R, Glidewell E, Pritchard C, Thomas R, Fraser C. Interventions to improve outpatient referrals from primary care to secondary care. Cochrane Database Syst Rev. 2008 Oct 8;(4):CD005471. doi: 10.1002/14651858.CD005471.pub2. http://www.ncbi.nlm.nih.gov/pubmed/18843691 (Accessed 13.4.13.)
2. Sittig DF, Ash JS, Zhang J, **Osheroff** JA, Shabot MM. Lessons from "Unexpected increased mortality after implementation of a commercially sold computerized

physician order entry system". Pediatrics. **2006** Aug;118(2):797-801. http://pediatrics.aappublications.org/content/118/2/797.long (Accessed 13.4.13.)
3. **Rosenthal DI**, Weilburg JB, Schultz T, Miller JC, Nixon V, Dreyer KJ, Thrall JH. Radiology order entry with decision support: initial clinical experience. **J Am Coll Radiol**. **2006** Oct;3(10):799-806. http://www.ncbi.nlm.nih.gov/pubmed/17412171 (Accessed 13.4.13.)
4. **Ip** IK, Schneider LI, Hanson R, Marchello D, Hultman P, Viera M, Chiango B, Andriole KP, Menard A, Schade S, Seltzer SE, **Khorasani** R. Adoption and meaningful use of computerized physician order entry with an integrated clinical decision support system for radiology: ten-year analysis in an urban teaching hospital. **J Am Coll Radiol**. **2012** Feb;9(2):129-36. doi: 10.1016/j.jacr.2011.10.010. http://www.ncbi.nlm.nih.gov/pubmed/22305699 (Accessed 13.4.13.)
5. **Raja** AS, Ip IK, Prevedello LM, Sodickson AD, Farkas C, Zane RD, Hanson R, Goldhaber SZ, Gill RR, Khorasani R. Effect of computerized clinical decision support on the use and yield of CT pulmonary angiography in the emergency department. **Radiology**. **2012** Feb;262(2):468-74. doi: 10.1148/radiol.11110951. Epub 2011 Dec 20. http://www.ncbi.nlm.nih.gov/pubmed/22187633 (Accessed 13.4.13.)

Talk 23: Innovations for improving Guideline use

Francesco Sardanelli, European Society of Radiology (ESR)

Francesco Sardanelli, EuroAIM Director
University of Milan School of Medicine, IRCCS Policlinico San Donato, Milan, Italy
f.sardanelli@grupposandonato.it

The role of radiologists and nuclear physicians in the authorship of systematic reviews on diagnostic and interventional imaging. Summary of a report of the EuroAIM working group

The relative delay of evidence-based medicine application to diagnostic and interventional imaging and the anecdotic finding of secondary studies on typical radiological procedures written by groups of "experts" not including radiologists or nuclear physician (imaging specialists) raised the idea to systematically investigate about the authorship of secondary imaging studies. This was carried out by a working group of the European Network for Assessment of Imaging in Medicine (EuroAIM) initiative.

We searched for systematic reviews (SRs) regarding diagnostic and interventional procedures, published in 2001-2010. Selection was initially based on Title/Abstract; only eligible SRs were fully assessed. SRs concerning procedures mainly performed by non-imaging specialists were excluded. Each SR was attributed to one of 11 subspecialties and one of 15 study categories.

Of 3,258 retrieved citations, 875 SRs entered analysis. A nearly-linear increase in the annual number of overall SRs was observed, from 26 in 2001 to 169 in 2010. A similar trend was observed for the SRs with at least one imaging specialists as an author, from 9 to 61, respectively. As a consequence, the rate of SRs with at least one imaging specialist as an author significantly (but more slowly) increased during the decade, reaching a peak of 44% in 2008; unfortunately, this trend declined in the last two years to 36%.

Neuroimaging was the most represented subspecialty (28%), followed by Gastrointestinal/Abdominal (12%). Diagnostic performance was the most represented category (41%), followed by Neurological Morphometry/Function (14%). Publication rate in imaging journals was 26%, from 6% (Pediatric) to 45% (General Oncology) and from 6% (Diagnostic Performance of a Non-imaging Test) to 75% (Technical Performance of an Imaging Test).

On average during the decade, only 20% of these secondary studies have an imaging specialist as first author, 19% as last author, 38% as an author in whatever position. Thus, there is an under-representation of imaging specialists in the authorship of SRs regarding imaging. This low proportion is alarming if we consider that imaging procedures mainly performed by non-imaging specialists were excluded from analysis. For a quick comparison, consider that the evaluation of first author's affiliation for 100 consecutive SRs published in 2010 attributable to a medical specialty of primary interest resulted in 85 SRs clearly authored by clinical experts of the investigated field (PubMed, accessed on September 3, 2012), while the rate of imaging specialists who authored in whatever position SRs on imaging in same year was only 36%

The balance between radiologists and nuclear physicians in the authorship resulted 138/39 (3.5:1) for the first position and 130/32 for the last position (4.1:1). Thus, radiologists overall contributed for about 80% of relevant positions of imaging specialists in authorship. Obviously, nuclear physicians contributed for 57% of relevant positions in authorship for General Oncology, as expected considering the relevance of radionuclide imaging in this field. Moreover, this almost balanced situation happens in the subspecialty with the far highest percentage of SRs with at least an imaging specialist in the authorship (88%).

The median journal impact factor of SRs published in 2009-2010 with at least one imaging specialist in the authorship (3.207) was lower than that of SRs without (4.327).

In conclusion, only 38% of SRs on imaging published from 2001 to 2010 has at least one radiologist or nuclear physician as an author. Imaging specialists are under-represented in the authorship of secondary evidence for procedures they daily perform and discuss with patients, referring physicians, healthcare providers, and decision makers. Policies to counteract this trend should be adopted.

Suggested reading

1. Sardanelli. Evidence-based radiology and its relationship with quality. In: Abujudeh HH, Bruno MA, eds. Quality and safety in radiology. New York: Oxford University Press, 2012: 256-290.
2. Evidence- Based Medicine Working Group. Evidence based medicine. A new approach to teaching the practice of medicine. JAMA. 1992;268(17):2420–2425.
3. Malone DE. Evidence- based practice in radiology: An introduction to the series. Radiology 2007;242(1):12-14.
4. Centre for Evidence- Based Medicine, Oxford University, England. Accessed July 9, 2010, at: http://cebm.net.
5. Sardanelli F, Hunink GM, Gilbert FJ, Di Leo G, Krestin GP. Evidence-based radiology: why and how? Eur Radiol 2010;20(1):1-15.

6. Medina LS, Blackmore CC. Evidence- based radiology: Review and dissemination. Radiology 2007;244(2):331–336.
7. Egger M, Smith GD, Altman DG. Systematic reviews in health care: Meta-Analysis in context, 2nd ed. London: BMJ Publishing group, 2009
8. EIBIR. European Institute for Biomedical Imaging Research. http://www.eibir.org/.

Talk 24: Innovations for improving Guideline use
Pete Cavanagh, The Royal College of Radiologists (RCR)

Dr. Pete Cavanagh
Vice President Royal College of Radiologists, UK
Peter.cavanagh@southwest.nhs.uk

Despite the significant effort and rigour involved in the production of referral guidelines in the UK there is still a significant challenge to ensure their effective usage. Although justification of a request by a radiologist has the potential for intervention after the referral has been made, the aim must be to influence decision-making by the referrer at the time of referral. A number of actions may facilitate this:

1. Ease of access at the point of referral

For example, the RCR has developed an App version of its guidance which is now available for smart phones and tablets. This could be further improved by being able to customise guidelines by user profile so that only the guidelines relevant to an individual practice are stored in active mode.

2. Decision support software

As electronic requesting becomes the norm, there is an opportunity to embed the guidelines into these software programmes, prompting the referrer to make the right decision. This will only work if it does not impede the ease of referral.

3. Education and training

Although there may be a role for education and training for the current and next generation of referrers, this in itself is unlikely to be a solution. Current Guideline documents contain hundreds of individual guidelines and although these will not all be relevant, it will require on-going re-enforcement. However more knowledge of this in the undergraduate curriculum should be pursued.

4. Incentives or Sanctions

In the current model of healthcare delivery in the UK there are opportunities to reimburse primary care doctors (general practitioners) for delivering certain aspects of quality care such as checking blood pressure and cholesterol screening. It is possible that this type of approach could be extended to correct use of imaging for certain conditions.

The reimbursement in England for healthcare is based on a system termed Payment by Results. The concept is that providers get paid for certain interventions, tests, or episodes of care. It may be possible to agree on withholding payment for certain diagnostic test that fall outside accepted guidance. However, the ‘policing’ of such a system could be difficult to implement across the whole of the guidelines.

5. Benchmarked information feedback

Referrers may be challenged to improve their referral patterns if accurate and timely feedback of their practice could be provided to them with indications of which guidelines may be relevant to their particular referral pattern.

It is unlikely that any one of these potential interventions will provide the solution in isolation. It would be important to test their potential benefit on a small scale where applicable to gain more information as to their potential benefit.

Talk 25: Innovations for improving Guideline use

Richard Mendelson, Western Australian Health Department

Prof. Richard Mendelson, Royal Perth Hospital, Perth, Western Australia
Richard.mendelson@health.wa.gov.au

There is evidence from our own studies and those of others that 'stand-alone' guidelines have limited utility in altering requesting behaviour among referring clinicians. Therefore, we are developing and evaluating an electronic request/decision support tool which integrates the academic content of DIP into the requesting process for diagnostic imaging so that imaging recommendations are a seamless part of the clinicians' work flow.

The application's opening electronic page shows the icons for the various organ systems. If, say neurological, is chosen the sub menu is displayed indicating the various neurological clinical scenarios. 'Suspected stroke' will be used to demonstrate the application.

Each scenario uses a flow chart or algorithmic format based on the content of the Western Australian "Diagnostic Imaging Pathways" (DIP) website.

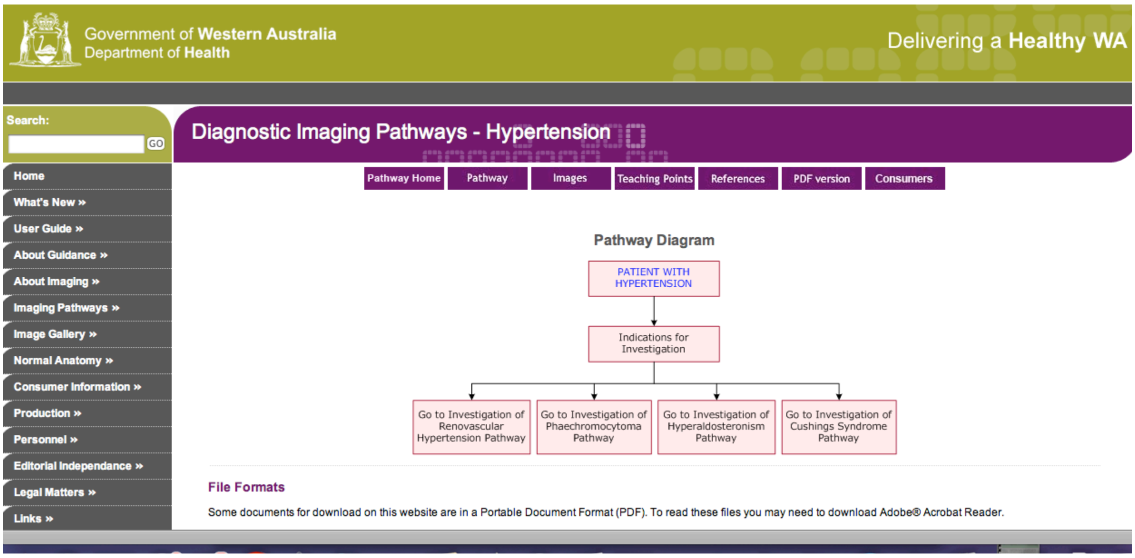

Each page has a drop down legend - showing the meaning of the abbreviations used in the flow chart, such as 'i' for general information 'ri' for radiology information and 'ci' for clinical information. Clicking on these icons either results in a succinct "pop-up" take-home

message or a link to more detailed information in DIP and the full functionality of the DIP website, including referenced narrative text, teaching points, the full DIP pathways, etc. The legend also indicates the meaning of the ionizing radiology icons used in the pop-up messages in terms of relative dose from 'none' to 'high'.
So, for example, returning to the stroke flowchart, there is some pop up clinical information indicating the role of imaging in this condition. Green boxes in the flow chart indicate the recommended imaging - in this case an urgent head CT scan. The user has the option of accepting or overriding the recommendations. If the recommendation is accepted, clicking the tick icon allows the user to request just that test or to add other tests. Perhaps in the case of suspected stroke a chest x-ray would be appropriate. Clicking 'request test' takes the user to the imaging service request form.

In a real-life situation the application would be linked to the Hospital Information System and Radiology Information System so all of the demographics and other information for that patient would be pre-populated on the form. However a patient can be selected manually from a drop-down work-list. Similarly, the radiology provider to which the request is directed can be automatically or manually entered.

The "clinical information" section of the request form has been populated with details that have been recorded by documenting the previous mouse clicks and how the point of request has been achieved. The referrer can sign the form electronically and, when ready, send the request electronically by internal or external secure messaging to the imaging provider. Obviously, the form that arrives at the other end can be customised by the practice. Some fields can be made mandatory, for example the "is the patient pregnant?" field.

Returning to the flow chart, the next step depends on the result of the initial CT Scan. Further imaging may be required contingent on the CT result and clinical situation.

Returning to the original scenario and considering that the referring doctor may believe that for his patient a MRI scan is more appropriate than a CT scan as the initial test and wishes to override the recommendation for CT. By clicking on 'override' the user is prompted to enter a justification for the override, which appears on the imaging request form. The user must then complete their choice of modality manually.

The imaging provider can customise the application so that some overrides can be allowed through, without consultation with an imaging specialist, while others may require consultation prior to acceptance. Of course, the application lends itself to extensive auditing of referrals, and in particular, the frequency and reasons for overriding of recommendations by individual clinicians.

Other examples of clinical scenarios use check boxes to determine whether imaging is required. For example, for ankle injury (accessed from the musculoskeletal trauma menu), initially the user is prompted to choose which side of the body has been injured and then is aided in applying the Ottawa ankle rule. Checking for example the 'inability to weight bear' leads to the process of requesting a plain ankle x-ray series. From then on this leads to a similar process we have already seen with CT in stroke. However if no parameters exist for indicating an x-ray, the user is informed of this, but has the option to override, but is required to provide justification for doing so.

In summary, the application is a potentially powerful audit tool. Of course, not all requests require decision support, for example a request for clavicle x-ray goes straight to the request form after the side of injury is selected.

Talk 26: Innovations for improving Guideline use
Martin Reed, The Canadian Association of Radiologists (CAR)

Dr. Martin H. Reed, Chair, Guidelines Working Group, Canadian Association of Radiologists
Department of Radiology, Children's Hospital, Winnipeg, Canada
MReed@exchange.hsc.mb.ca

The Canadian Association of Radiologists (CAR) referral guidelines were published in both English and French versions in booklet form in 2005. The guidelines were also made available on the Internet through password protected websites including the CAR website, the CMA Infobase and the website of the College of Family Physicians. A PDF version was also made available on CD. The guidelines were quite widely circulated and the first English printing of one thousand copies was sold out and a second printing had to be made.

However, the CAR also recognized that making guidelines available in all these formats did not guarantee their utilization. Believing that integrating the guidelines into the physician's workflow would be the best method of ensuring their uptake and utilization, a software company, Medicalis, based in Waterloo, Ontario, was approached. They had developed a computerized order entry (CPOE) system for diagnostic imaging and they were willing to incorporate the CAR guidelines into that CPOE. Two projects were undertaken to assess the effectiveness of incorporating the CAR guidelines into a CPOE. The initial project was carried out at the Children's Hospital in Winnipeg between October 2006 and August 2007. Seventy-seven pediatricians and twenty-seven pediatric residents and Fellows participated in that project. Almost nine thousand orders were placed using the software (Table) and of these approximately 11% were considered inappropriate according the CAR referral guidelines. However, the advice to change the order was only accepted in 2% of cases. The second project was carried out with a group of family practitioners at the Steinbach Family Medical Centre between November 2008 and February 2009. This was a smaller project with only nine hundred orders being placed using the software (Table). A similar percentage of orders (14%) were considered inappropriate according to the CAR referral guidelines, but in this case the advice to change the order was accepted in 25% of cases.

Table: Results of studies

**Project**	**Total orders**	**Inappropriate orders**	**Advice accepted**
Children's Hospital	8757	957 (10.9%)	19 (1.9%)
Steinbach	904	123 (13.6%)	31 (25.5%)

A third project was undertaken at the Children's Hospital in Winnipeg between February 2010 and July 2012 to try to use several interventions to improve physicians' ordering patterns. The original Medicalis software had been used on a regular basis at the Children's Hospital up until the beginning of this new project. Unfortunately, however, Medicalis had changed their software in the interval and for various reasons the new software had to be implemented in order to carry out the new project. This new software had been designed without consultation with users at the Children's Hospital and it turned out to be very different from the software that they were used to using. It was also found to be less user friendly and slower to use. Numerous problems were encountered in attempting to improve the software from the users' point of view, to integrate it into the Radiology Information Management System at Children's Hospital and to improve its speed. Because of the rather limited funding and other priorities for both Medicalis and the IT Department at the Children's Hospital all these problems were never completely solved during the lifetime of the project. As a result the interventions could not be tested and assessed.

The CAR believes that a number of lessons have been learned from the various projects that the organization has undertaken to assess the value of incorporating referral guidelines into a CPOE for diagnostic imaging. These lessons include:

- Any software program incorporating decision support into a CPOE has to be simple, fast, and transparently integrated into existing electronic systems.
- Any project designed to introduce CPOE with decision support must have the resources to allow adequate time for the project and to provide adequate IT support for the project.
- These projects cannot be simply implemented by administration. They require leadership by physicians respected in the community.
- Initially the software should be tested with a group of interested physicians who have IT expertise and can provide constructive advice about any deficiencies in the software.
- Because decision support does to some extent interfere with physicians" workflow, only a limited number of important referral guidelines should be integrated into the software. The decision about which guidelines should be used should be made in consultation with both referring physicians and radiologists on site.

The CAR is committed to continuing to improve and update their referral guidelines, to encourage their use as widely as possible in the physician community in Canada and to study the best methods of incorporating referral guidelines into CPOE's and to assess the effectiveness of this approach to implementing referral guidelines.

Talk 27: Innovations for improving Guideline use

Michael Bettmann, American College of Radiologists (ACR)

Michael Bettmann, MD, FACR, FAHA
Chair, ACR Appropriateness Criteria Oversight Committee, Co-Chair, ACR Task Force on Decision Support

Challenges to the use of imaging in the US

- Inappropriate use, for many reasons

- Attempts at external controls add cost, delay-
    - Radiology Benefit Managers,
    - Insurance company hurdles and constraints
- Guidelines in format usable in Electronic medical records, EMRs are sparse, poor and/or hard to use.

Challenges to the ACR Appropriateness Criteria

- Usability of guidelines: format, scope
- Availability in electronic format
- Buy-in: Users individually,
    - Health-care organizations,
    - EMR vendors,
    - Other medical societies
- Process for revision, addition, validation

Current ACR Effort: Creation of a useable imaging clinical imaging decision support tool

- ACR Select-collaboration with National Decision Support Company, NDSC
- Content is ACR Appropriateness criteria, supplemented as needed
- Content as a fixed data-base will be sold by NDSC
- Content owned by ACR. Cannot be altered by users

Aim of ACR Select

- Serve as on-line CDS tool, to improve the appropriate use of imaging-decrease inappropriate exams, decrease unnecessary radiation exposure
- Through feedback from users, continual improvement of the coverage, usability and validity of the ACR AC
- Through analysis of use by individuals, groups and healthcare organizations, collect and analyze data on utilization and utility of imaging
- Eliminate unnecessary hurdles

ACR Select

- Sold to vendors of CPOE/ EMR systems
- Sold directly or secondarily to health care organizations or Decision Support System, DSS creators
- Key considerations are:
    - methodological soundness,
    - ongoing feedback with users and vendors,
    - sound, web-based IT format
